# The Role of Hydrogen Bond Donor on the Extraction of Phenolic Compounds from Natural Matrices Using Deep Eutectic Systems

**DOI:** 10.3390/molecules26082336

**Published:** 2021-04-17

**Authors:** Duarte Rente, Alexandre Paiva, Ana Rita Duarte

**Affiliations:** 1 LAQV, REQUIMTE, Departamento de Química da Faculdade de Ciências e Tecnologia, Universidade Nova de Lisboa, 2829-516 Caparica, Portugal; d.rente@campus.fct.unl.pt (D.R.); abp08838@fct.unl.pt (A.P.); 2 Des Solutio, Avenida Tenente Valadim, nº 17, 2ºF, 2560-275 Torres Vedras, Portugal

**Keywords:** deep eutectic systems, phenolic compounds, antioxidants, natural matrices, hydrogen bond donor, extraction

## Abstract

Recently, deep eutectic systems (DESs) as extraction techniques for bioactive compounds have surfaced as a greener alternative to common organic solvents. In order to study the effect of these systems on the extraction of phenolic compounds from different natural sources, a comprehensive review of the state of the art was carried out. In a first approach, the addition of water to these systems and its effect on DES physicochemical properties such as polarity, viscosity, and acidity was investigated. This review studied the effect of the hydrogen bond donor (HBD) on the nature of the extracted phenolics. The effects of the nature of the HBD, namely carbon chain length as well as the number of hydroxyl, methyl, and carbonyl groups, have shown to play a critical role in the extraction of different phenolic compounds. This review highlights the differences between DES systems and systematizes the results published in the literature, so that a more comprehensive evaluation of the systems can be carried out before any experimental trial.

## 1. Introduction

Most phenolic compounds are secondary metabolites [1], found in a wide number of plants, fruits, and vegetables, that are responsible for plant growth and development while also displaying sensorial, structural, and defensive properties [2,3]. From a chemical point of view, they are characterized by the presence of one or more hydroxyl groups bound to an aromatic ring [2,4,5]. Depending on the number of carbon atoms, they can be classified into several groups, including phenolic acids (hydroxybenzoic and hydroxycinnamic acids), flavonoids (flavones, flavanones), and condensed tannins (lignin) [2]. Phenolic compounds have strong antioxidant activities [6,7], granting them desirable biological and pharmacological properties that include but are not limited to anti-inflammatory, neuroprotective, antimutagenic, and anticarcinogenic properties [6,7,8,9]. For these reasons, phenolic compounds have found a wide range of applications in several areas of interest such as cosmetics, food additives, nutraceuticals, and pharmaceuticals [4,10,11]. Phenolic compounds can be obtained either via synthetic processes or from natural sources. The synthesis of phenolic compounds is done through organic synthesis, which usually requires the preparation of intermediary species and multiple reaction steps. These individual steps require long reaction times, high temperatures, the use of toxic and harmful reagents, and the need for further purification in order to remove unwanted by-products and waste material, resulting in low yields [12]. For natural sources such as plant and vegetable matrices, an extraction step is required to obtain the desired compounds. There are a wide variety of natural matrices, however; wastes from the agricultural and food industries [13] such as wine lees and olive pomace [14,15,16,17] can be used as source materials, thus reducing the environmental impact of the industries. The extraction step is crucial, since these compounds can be degraded and therefore have their activity compromised by the processing methodologies employed.

The extraction of phenolic compounds from solid plant matrices can be carried out using either conventional methods or alternative methods [6]. Conventional extraction methodologies include solid/liquid and Soxhlet extractions, maceration, and percolation, which are associated with long extraction periods and high temperatures that can lead to phenolic thermal degradation and low yields, as well as the use of extensive amounts of toxic and harmful organic solvents and the production of high quantities of waste products [4,7,18].

**Figure 1:**
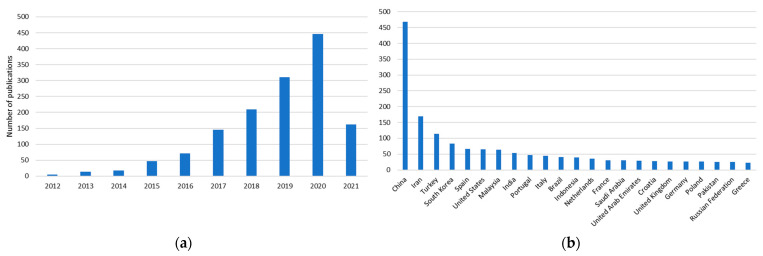
(**a**) Number of articles published since 2012 regarding the use of DES as extraction solvents; (**b**) geographical distribution of the countries where the research concerning DES was conducted. (Data on scopus.com using the keywords “deep eutectic solvents extraction”, accessed on 18 March 2021).

Alternative extraction methodologies like enzyme-assisted extraction, ultrasound-assisted extraction (UAE), microwave-assisted extraction (MAE), subcritical and supercritical extractions, and high-pressure-assisted extractions [19,20,21] can be used to overcome some of the problems associated with conventional extraction methods, since they offer lower extraction times, higher yields, and use of lower amount of solvents, whether they be green or organic solvents [6]. However, despite the advantages modern methods may have over conventional methods, both groups of methods have problems regarding solvent toxicity, thermal instability, polarity, solubility, and poor selectivity [4], as well as the use of specialized and high-cost equipment [6,22]. Furthermore, common organic solvents such as methanol, ethanol, and acetone [6,23] are highly volatile and flammable, making them health and environmental hazards [24,25]. Considering that the applications of most of the extracted phenolic compounds are in the food, nutraceutical, and pharmaceutical industries, where the use of organic solvents such as ethanol, methanol, and dichloromethane is heavily regulated [26], there is a need to find greener and safer options that are harmless for human consumption. Recently, deep eutectic solvents have been gaining a lot of attention as green extraction solvents. As it can be seen in Figure 1, research on DES extractions has grown exponentially since 2012, with places like China, Iran, and Turkey producing the most research on the topic.

## 2. Deep Eutectic Systems

Deep eutectic systems are the result of the complexation of a hydrogen bond acceptor (HBA) and a hydrogen bond donor (HBD), which are usually naturally occurring, and biodegradable compounds. For this reason, it is expected that DES will be biodegradable and present a lower toxicity when compared to other solvents. This novel class of solvents has a set of advantageous properties, such as low melting points, low volatility, nonflammability, low vapor pressure, polarity, chemical and thermal stability, miscibility, and high solubility [4,10,18,27,28], properties that make DESs a safer alternative to conventional extraction solvents. Additionally, they can be produced at low costs and high yields without the need for further purification steps [18,24,25,27]. Since DESs are formed through intermolecular interactions such as hydrogen bonding, there is no chemical reaction involved, hence there is no production of secondary compounds. As such, every atom in the system is used, meaning that DES production should have 100% atom economy. Similarly, since DESs result from the molecular interactions of two or more compounds, no waste is usually produced, meaning that DESs have a very low E-Factor. The intermolecular interactions, mainly hydrogen bond interactions, between the HBA and the HBD are responsible for the physicochemical characteristics of the DES [10,29], which means that by changing either the HBA/HBD ratio or one of the components, the DES can be specifically tailored for different applications. This is a major advantage in terms of achieving desirable properties and improving the extraction efficiency [25,27]. The preparation of a DES can be done either via the stirring and heating method [22,29,30,31], where the HBA and HBD are mixed in the desired ratio at high temperatures until a clear liquid forms, or by the freeze-drying method [22,25,29], where the HBA/HBD mixture is dissolved in water, brought to −80 °C, and lyophilized until a constant weight is reached. With the exponential growth in this field of research and the versatility of DESs, a question worth answering is whether the influence of the HBD plays a role in the extraction selectivity of phenolic compounds.

DESs are able to dissolve a wide range of compounds, both polar and nonpolar, making them a desirable extraction medium for phenolic compounds [16]. Furthermore, they are also able to form hydrogen bonds with phenolic compounds, improving their dissolution and extraction ability [32,33]. Extractions of phenolic compounds using DESs are often solid/liquid extractions in which a liquid solvent, the DES, is used to extract compounds of interest from a solid plant matrix. There are several extraction techniques [34], but the most used are heat stirring (H/S), ultrasound-assisted extraction (UAE), and microwave-assisted extraction (MAE). While the use of MAE is a more efficient method, producing higher yields in relatively shorter times, the uses of UAE and H/S are more prevalent, since the equipment required to perform these extractions, i.e., a stirring plate or an ultrasound bath, is easily accessible and less expensive than microwave equipment and offers better potential for large-scale processes [6,35].

Over the last few years, several review articles have been published relating to deep eutectic systems, as well as their use in the extraction of polyphenolics. While the review of Clarke et al. [36] is geared towards green and sustainable solvents in general, such as ionic liquids, DES, liquid polymers, and supercritical CO_2_, important considerations regarding toxicity are still brought up, since even if the components of a DES have low toxicities or are nontoxic by themselves, the same is not always true when they are mixed together [37,38]. In more specific cases regarding eutectic systems, the work by Liu et al. [29] is a comprehensive review related to the fundamentals of natural deep eutectic systems (NaDESs), mainly regarding how the hydrogen bonding interactions between the HBD and the HBA, studied by NMR and FTIR, relate to the system’s physicochemical properties, such as conductivity, viscosity, polarity, solubility, stability, and biocompatibility, while also discussing potential applications of NaDESs as extraction media, chromatographic media, and biomedical carriers. Works from Bubalo et al. [39], Cunha et al. [34], and Zainal-Abidin et al. [4] discuss the fundamentals of DESs while exploring the application of DESs in the extraction of phenolic compounds from natural matrices. The work of Bubalo et al. [39] is focused on the comparison of different extraction green solvents such supercritical CO_2_, subcritical water, and NaDESs, while Cunha et al. [34] deal with different extraction methods, such as ultrasound-assisted extraction or microwave-assisted extractions. Zainal-Abidin et al. [4] reviewed and, more recently, Fernández et al. [40] focused on the extraction of different plant metabolites and how their physicochemical properties may influence the extraction efficiencies of DESs. All of the previously mentioned articles show the versatility of DESs in the extraction of different bioactive compounds from natural matrices.

**Figure 2:**
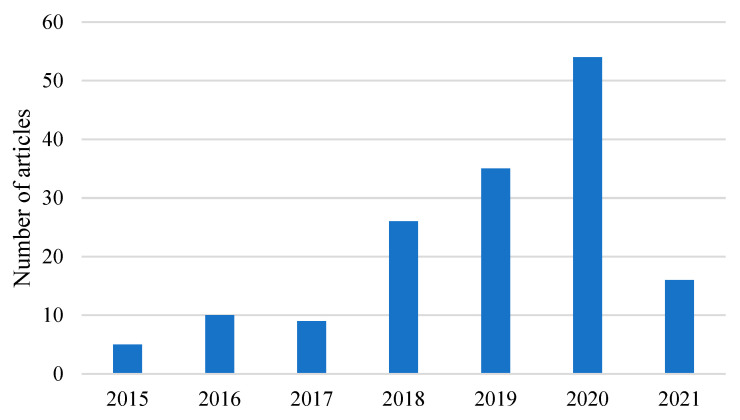
Number of articles published in the last six years regarding the extraction of phenolic compounds such as phenolic acids and flavonoids. (Data on scopus.com using keywords “deep eutectic solvents phenolic extraction”, accessed on 18 March 2021).

While building on the ideas in some of the reviews discussed, the intent of this work was to study the extraction selectivity of different phenolic acids and flavonoids using DESs, by trying to find a correlation between the functional group of the HBD and the chemical structure of the extracted compounds. To do so, a review of the state of the art was conducted. Since 2015, around 155 articles on the extraction of phenolic compounds using DESs have been published (Figure 2).

**Figure 3:**
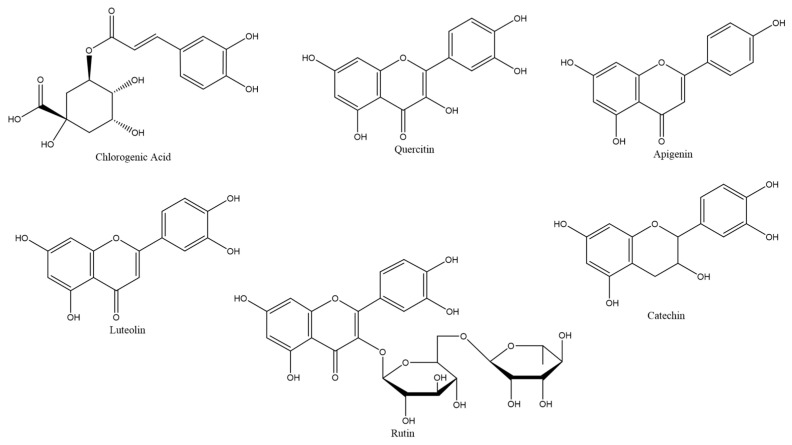
Chemical structures of commonly found phenolic compounds.

In a first approach to the available data, articles were selected if they presented colorimetric and/or HPLC data regarding the extraction of different phenolic acids or flavonoids using a DES and a reference organic solvent such as water, ethanol, and methanol. Due to the focus on phenolic acids and flavonoids, articles pertaining to the extraction of other compounds such as terpenes, lignocellulosic compounds, or metals from plant and vegetable matrices were not considered. Using the described criteria, around 28 articles were found for study, which comprised 18% of articles published in the last six years. The articles reviewed showed that at least 86 individual chemical compounds were identified (Figure 3) and extracted from 32 different plant matrices.

Regarding the solvents used, 173 DESs with different HBA/HBD ratios were identified, with 93 of these being unique combinations of an HBA/HBD. From the 173 combinations used, 140 used an ammonium salt as the HBA, such as choline chloride (105 systems) or betaine (35 systems), which accounted for 80% of the analysed DESs. Despite its wide use, the European Commission for Cosmetic Ingredients [26] has banned choline chloride use due to its irritant properties. As a possible alternative, betaine has been proposed [33,41]. Aroso et al. [41] showed that using the same HBD, DESs based on betaine proved more difficult to prepare, often requiring the use of water as a ternary component. The structural differences between choline chloride and betaine will lead to different interactions with the bioactive compounds extracted, either hindering or promoting the extraction yield. Fanali et al. [42] tested similar DESs based on choline chloride and betaine in the extraction of spent coffee grounds. They found that in this case, betaine-based systems had higher extraction yields than their choline-chloride-based counterparts. While this shows that the HBA has an influence in extraction yields, for the purpose of this work the effect of the HBA was not considered, with greater importance given to the effect of the HBD.

**Figure 4:**
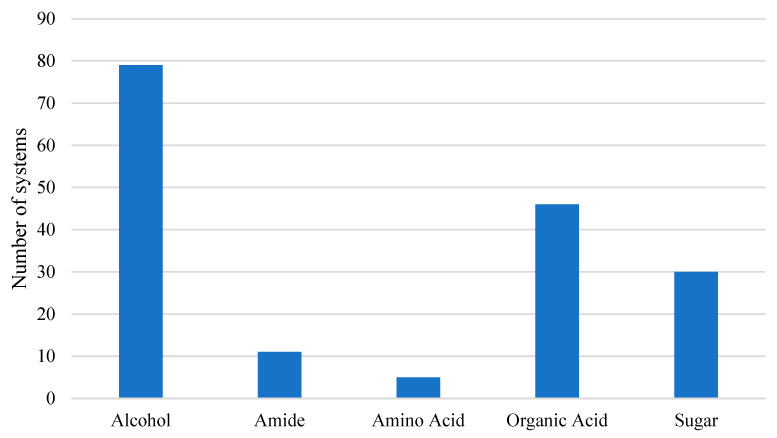
Distribution of the different hbds studied, organized by funtional group with alcohol-, organic-acid-, and sugar-based systems being the most common.

The HBDs of the reported systems can be divided according to their functional groups (Figure 4), with systems based on alcohols (79 systems), organic acids (46 systems), and sugars (30 systems) being primarily used.

Thirty-nine individual compounds were used as HBDs to form DESs, with compounds such as glucose (13 systems), lactic acid (14 systems), citric acid (6 systems), sucrose (8 systems), and glycerol (8 systems) being some of the most used. Choline chloride was one of the first HBAs reported by Abbot et al. [31] in 2004 and systems using it, alongside some of the HBDs mentioned, have been studied and characterized [27,28,30,31,34,43]. Since the systems are described in the literature, their use in new applications such as extractions can be considered as a starting point or a comparison term before testing new systems.

While it is important to study the extraction conditions used, such as extraction time and temperature, these are often set between 45 to 60 min and 40 °C to 65 °C and will not be discussed here.

Besides the experimental conditions used during extraction, there is also a need to take into consideration the physicochemical properties of the DES. Viscosity and polarity are properties that surely play a role in the type of bioactive compound extracted; however, this is information that is not always readily available. While scarce, authors such as Dai et al. [32] have published experimental data regarding some physicochemical properties and, recently, Haghbakhsh et al. [44,45], Bakhtyari et al. [46], and Taherzadeh et al. [47] developed mathematical models to describe these properties. The problem with this information relates to the fact that it is obtained for pure DESs and it is common practice to add water to the systems (Figure 4), which will modulate the properties in each individual case. As such, it is not possible to accurately explore the effects of viscosity and polarity on the types of bioactive compounds extracted.

**Figure 5:**
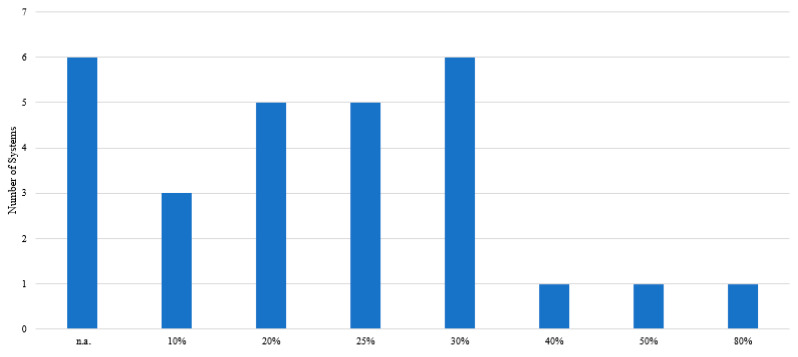
Amount of water used to aid extraction of phenolic compounds from different natural matrices, with n.a. Meaning information not available or that water was not added.

High viscosities, which are a characteristic of most DESs, are a drawback of these systems, since they act as a hindrance in mass transfer phenomena and thus lower the extraction efficiencies of the DES [48]. Viscosity is a physicochemical property highly dependent on temperature, meaning that upon increasing the temperature by even a few degrees, the viscosity of the system is reduced drastically and hence, mass transfer phenomena is improved. Nonetheless, as some phenolic compounds are thermolabile, an increase in temperature may not always be the solution. This drawback can also be overcome by the addition of water to the systems [22,25,49]. Figure 5 shows the amount of water which has been added to the DESs used to perform extractions. The water content of the system varies between 10% and 80%, with the most commonly used being 20% to 30% *w*/*w*.

The amount of water in a DES has been a topic of discussion due to the possibility of water disrupting the intermolecular interactions between the HBA and the HBD. Recent studies [50,51,52] have shown that water is able to form hydrogen bonds with the HBA and the HBD, and thus help to modulate DES properties. However, as the amount of water in the system increases, the intermolecular interaction between the HBA and the HBD weakens until the mixture becomes a solution, with Zhekenov et al. [51] suggesting that this limit is at 50% mol. fraction of water.

The addition of water plays a very important role in DES properties such as viscosity, polarity, and acidity and, consequently, on the extraction yield of the DES.

In one of previous study, Mansur et al. [53] found that the addition of water to a DES significantly improved the extraction yields of flavonoids when compared with extractions where no water was added. They also studied the effect of different amounts of water content in the DES, from 20% to 80%, and concluded that despite the addition of water improving the extraction of flavonoids when compared with a DES with no water, 20% water provided the best extraction results. Besides affecting the viscosity of the systems, the addition of water will also change other physicochemical properties, such as polarity and acidity of the different solvents, which in turn also play an important role in the results obtained. Regarding polarity, Xu et al. [21] showed that fine-tuning the system polarity can be used to improve the extraction yield of different flavonoids. In their work, they performed multiple extractions of citrus flavonoids using choline-chloride-based systems with different HBDs. By keeping the HBA constant, the polarity of the systems was made dependent on the different HBDs used and polarity was measured using the *n*-octanol/water partition coefficient of the HBD. The authors found that systems with lower polarities extracted higher amounts of citrus flavonoids such as hesperidin, which have low polarities. The higher extraction efficiencies can be explained by the similar polarities between the DES and phenolic. Besides playing an important role in the viscosity and polarity of DESs, the addition of water can also be used to tune DES acidity. DESs are a mixture of compounds complexed by intermolecular interactions, therefore, there are no free protons in the liquid media; thus, measuring the system’s acidity using a conventional pH electrode does not render a value with physical meaning. Abbott et al. [54] published work showing a spectrophotometric method that could be used to measure DES acidity; however, the technique described is not straightforward. Nonetheless, upon adding water to a DES, which will be free and not part of the DES structure, there will be protons in solution and therefore the pH can be measured using a conventional electrode.

Postprocessing and isolation of the active compounds after extraction is not an easy task. It is difficult to separate the DES from the extracted phenolics due its low vapor pressure and usually high viscosity. As such, most purification methods involve the use of membrane and antisolvent processes which can be expensive [16,33]. An alternative that has recently been gaining attention [22] is the use of tailored-made DESs that can both extract phenolic compounds from plant matrices and act as stabilizing agents, being incorporated into the final product, namely for further applications in cosmetic and pharmaceutical formulations. In this sense, the idea is to formulate a bioactive composition that can be incorporated into a final product. Regarding the stability of bioactives, Dai et al. [55] showed that flavonoids such as quercetin are able to form hydrogen bonds with a DES made of choline chloride and sucrose, which increased their stability in the DES. Other researchers [55,56] have also proven, following the degradation kinetics, that a DES can be used to stabilize phenolic compounds.

## 3. Discussion

As previously discussed, many different DESs have been used for extraction; however, a clear relationship between the DES and the nature of extracted phenolic compounds is still difficult. This work sought to shed light on the issue by studying the type of HBD used and their effects on different phenolic compounds by conducting a review of the state of the art. Due to the large amount of information available, several selection criteria were defined to refine the information collected. The selection criteria were defined as follows:

- Systems were selected as data points if the DES was a binary mixture;
- Ternary eutectic mixtures were only considered if the third component was water;
- If a DES was shown to be able to extract more than one compound from the same plant matrix, then a data point was considered for each compound extracted. For example, Viera et al. [57] reported, among others, a choline chloride:citric acid system (2:1) that was able to extract three types of flavonoids, querticin-3-*O*-glucoside, querticin-*O*-pentoside, and 3-*O*-caffeyolquinic acid, from walnuts and therefore three data points were considered;
- Data points were only selected if there data were reported on the yield of extraction using the DES and one of three conventional extraction solvents: water, ethanol, or methanol, which were used for comparison purposes.

**Figure 6:**
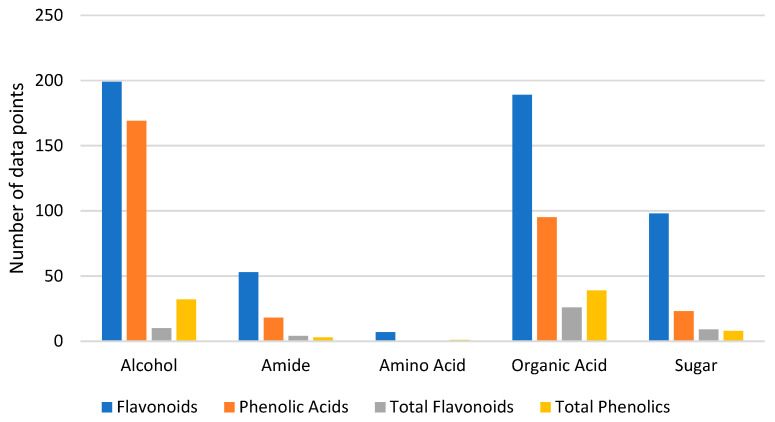
Distribution of the different compounds extracted, grouped by the functional group of the HBD and the nature of the phenolic extracted.

Since the physicochemical properties such as density, viscosity, polarity, pH, etc. of DESs are a result of the molecular interactions [58] between the HBA and the HBD, and a wide range of HBDs have been used for extraction, the functional group of the HBD was used as a reference to interpret the results reported in the literature, as it ultimately influences the final physicochemical properties of the systems. In a first approach, the DES were grouped by the HBD functional group (alcohol, amide, amino acid, organic acid, and sugar), and then each group was divided according to the nature of the extracted compounds (flavonoid, phenolic acids, total flavonoids, and total phenolics; Figure 6).

Considering the functional group of the HBD and the type of phenolic extracted, we observed that flavonoids have mostly been extracted using HBDs based on organic acids, alcohols, and sugars, while phenolic acids have been mostly extracted using alcohol- and organic-acid-based HBDs. To better understand the role of HBDs, the extraction yields of the DES were compared with the extraction yields of common organic solvents to determine the extraction efficiency (EE) of the DESs. Extraction efficiencies were defined as the ratio between the extraction yield of the DES and the extraction yield of the organic solvent used as control Equation (1), when data for both solvents were reported by the authors. The data were selected for the optimized parameters when calculating the EE.

(1) $$Extraction\;Effiency=\frac{DES\;Extraction\;Yield}{Organic\;Solvent\;Extraction\;Yield}$$

It is important to note that since the EE is the ratio between DES yields and organic solvent yields, in studies with an EE lower than 1, the DES had a lower extraction yield than the organic solvent. The following tables present the EE values of different target compounds extracted using DESs, using HBAs based on ammonium salts such as choline chloride grouped by the functional group of the HBDs, extraction technique, and natural matrices. Due to the high number of data points, the information was condensed by the phenolic extracted. Table 1 shows the effect of different HBDs on the EE of flavonoids from several matrices using water as the comparison organic solvent, while Table 2 and Table 3 present similar information using ethanol and methanol respectively as the extraction solvent for comparison. Unless otherwise mentioned, the DESs presented in the tables are based on choline chloride. Natural matrices were found to be in the form of a freeze-dried powder before use.

When using water as a control (Table 1), an overall improvement in EE can be seen, while the improvement is not as noticeable when using ethanol and methanol. This effect may be related to solute–solvent interactions, particularly the effect of solvent polarity in extraction. While this is an important factor, there are other factors to take into consideration that are also observable when using ethanol and methanol as comparison solvents (Table 2 and Table 3). In the case of alcohol-based HBDs, there are two main factors that influence EE: carbon chain length and the number of hydroxyl groups. Carbon chain length appears to have an inverse relationship with EE, since a decrease of the number of carbons in the chain (1,4-butanediol < 1,2-propanediol < ethylene glycol) increased the EE of compounds such as apigenin, hesperidin, and luteolin. On the other hand, looking at the effect of sugar alcohols in the same compounds, an increase of EE can be seen with an increase of the number of hydroxyl groups present, as in glycerol < xylitol < sorbitol. When working with amide-based systems, a clear relationship is harder to see, since EE values are similar; however, a higher degree of methylation appears to improve EE, especially in the case of hesperidin. The major effect of organic-acid-based systems in the extraction of flavonoids seems to be tied to the degree of carboxylation of the HBD. Looking at the extraction of quercetin glycosides, Vieira et al. [57] showed that the EE decreases with increasing degrees of carboxylation. This trend can also be observed in the extraction of hesperidin from Nobis tangerine; however, in the case of luteolin and apigenin, di and tricarboxylic acids produced an increase of the EE. The main difference could be attributed to the fact that hesperidin, quercetin-3-*O*-glucoside, and quercetin-*O*-pentoside are glycosides, while luteolin and apigenin are aglycones. As such, it can be inferred that when targeting aglycones, a higher degree of carboxylation may be desired, while the reverse is favored when trying to extract glycosides. Using sugar based HBD’s, the complexity of the sugar behaves like the carboxylation degree in organic acids. In this case simpler sugars such as fructose and glucose show that the use of DES increases the EE of different flavonoids.

A similar approach was taken to look for correlations between the functional group of the HBD and the extraction of phenolic acids. These correlations can be found in Table 4, Table 5, and Table 6, with water, ethanol, and methanol as the respective comparison solvents.

Looking at the effect of the HBDs in the extraction of phenolic acids, similar trends regarding alcohol chain length, hydroxylation, amide methylation, organic acid carboxylation, and sugar complexity can be observed. Despite the existence of outliers, alcohol-based systems have increased extraction efficiencies. This increase can be related to the systems’ acidity. When the HBA remains constant, the acidity of the DES is determined by the HBD. Since alcohols generally have higher pKa [78] values than organic acids, systems formed with these compounds will be more basic in nature. As such, it is possible that the phenolic acids have acid–base interactions with the alcohol-based systems, promoting their extraction.

Another interesting research avenue is the application of the Hildebrand Hansen solubility parameters, which measures the solute–solvent interactions between the DES and the bioactive compounds. While data are still limited, Salehi et al. [79] used molecular dynamics to shown that DESs with choline chloride and urea, ethylene glycol, glycerol, malonic acid, and oxalic acid have high solubility parameters, indicating that these can be considered polar solvents.

## 4. Conclusions

In recent years, research into DESs and their application for the extraction of phenolic compounds has grown exponentially. This growth can be attributed to their ease of preparation and use, as well as the green and safer properties inherent to DESs. When used as extraction media, three main extraction methodologies have been used, namely UAE, U/S, and MAE, with UAE and H/S being the most used. The amount of water in the system has been shown to play a critical role in the tuning of different physicochemical properties, namely viscosity, polarity, and acidity. Using the HBD functional group as a starting point for comparison purposes, this review evaluated the extraction efficiencies reported in the literature as a comparison tool to understand the effects of the HBD on the types of phenolic compounds extracted and the yield of extraction. The extracted compounds fell into under two major phenolic families, phenolic acids and flavonoids, mainly anthocyanins, flavones, and flavonols. Taking into consideration the nature of the HBDs and the type of phenolic extracted, some correlations can be drawn regarding the effectiveness of the HBDs. When using alcohol-based systems, small carbon chains and a high degree of hydroxylation are desirable, while a high degree of methylation is preferable in amide-based systems. In organic-acid-based systems, there are two factors which play a major role in the extraction yield: the degree of carboxylation and whether the extracted compound is an aglycone or not. If the extracted compound is an aglycone, a higher degree of carboxylation is more desirable, while the reverse is true for glycosides. If the desired compound is a phenolic acid, alcohol-based systems, which are more basic in nature, are desired, with the same rules previously discussed still applying. In the future, the influence of critical factors such as amount of water, pH, and polarities, which have not yet been reported in the literature, could also help develop our understanding of the effect of HBDs in the extraction efficiency and selectivity of these solvents towards particular classes of phenolic compounds.

## Figures and Tables

**Table 1:** Correlation between the natural matrix, extraction method, and DES based on ammonium salts (choline chloride and betaine) and the effect of different HBDs on the extraction efficiency of different flavonoids compared with water extractions.

Natural Matrix	Extraction Method	Extracted Compound	Hydrogen Bond Donor	Water EE	Reference
**Alcohol-based systems**					
Virgin Olive Oil	H/S	Apigenin	1,4-Butanediol; Glycerol; Xylitol; 1,2-Propanediol;	40; 40; 43.33; 50.00;	Garcia et al. [16]
		Luteolin	1,4-Butanediol; Glycerol; Xylitol; 1,2-Propanediol;	8.50; 8.50; 9.25; 10.13;	
*Lycium barbarum*	UAE	Myricetin	Ethylene Glycol; 1,2-Propanediol; Glycerol;	1.00; 1.25; 1.25;	Ali et al. [59]
Buckwheat Sprouts	UAE	Quercetin-3-O-robinoside	Glycerol; 1,4-Butanediol; 1,2-Propanediol; Ethylene Glycol; Triethylene Glycol;	0.68; 0.82; 0.86; 0.97; 1.46;	Mansur et al. [53]
*Lycium barbarum; Buckwheat sprouts*	UAE	Rutin	Xylitol; Glycerol; Glycerol; 1,2-Propanediol; Ethylene Glycol; Ethylene Glycol; 1,4-Butanediol; 1,2-Propanediol; Triethylene Glycol;	0.50; 0.75; 0.80; 1.00; 1.00; 1.09; 1.13; 1.24; 1.48;	Ali et al. [59]; Mansur et al. [53]
Platycladi Cacumen	UAE	Myricitrin	Ethylene Glycol ^a^; Ethylene Glycol ^a^;	1.42; 1.43;	Zhuang et al. [60]
*Lycium barbarum*	UAE	Morin	Glycerol; 1,2-Propanediol; Xylitol; Ethylene Glycol;	0.73; 1.09; 1.23; 2.73;	Ali et al. [59]
Buckwheat Sprouts	UAE	Vitexin	Glycerol; 1,2-Propanediol; Ethylene Glycol; 1,4-Butanediol; Triethylene Glycol;	0.83; 0.97; 1.13; 1.24; 1.76;	Mansur et al. [53]
		Orientin	1,2-Propanediol; Glycerol; 1,4-Butanediol; Ethylene Glycol; Triethylene Glycol;	0.64; 0.74; 0.87; 1.04; 1.44;	
*Platycladi cacumen*	UAE	Quercitrin	Ethylene Glycol ^a^; Ethylene Glycol ^a^;	1.51; 154	Zhuang et al. [60]
Buckwheat Sprouts	UAE	Isovitexin	Glycerol; 1,2-Propanediol; Ethylene Glycol; Urea; 1,4-Butanediol; Triethylene Glycol;	1; 1.17; 1.34; 1.48; 1.45; 1.95;	Mansur et al. [53].
		Isoorientin	1,2-Propanediol; Glycerol; 1,4-Butanediol; Ethylene Glycol; Triethylene Glycol;	0.89; 0.90; 1.12; 1.22; 1.69;	
*Nobis tangerine*	UAE	Hespiridin	Xylitol; Malic Acid; Maltose; 1,2-Propanediol; Glycerol; Propanedioic Acid; Sorbitol; Levulinic Acid; Oxalic Acid; Lactic Acid; Ethylene Glycol;	5.10; 5.25; 5.35; 5.55; 5.60; 6.15;	Xu et al. [21]
Wild Rice Powder	UAE	Procyanidin B1	Glycerol; 1,4-Butanediol;	1.56; 1.33;	Zeng et al. [61]
Spent Coffee	UAE	Total Flavonoids	Sorbitol; Glycerol; Ethylene Glycol; 1,4-Butanediol; 1,6-Hexanediol;	0.42; 0.96; 1.15; 1.42; 1.58;	Yoo et al. [14]
Wild Rice Powder	UAE	Quercetin	Glycerol; 1,4-Butanediol;	1.15; 1.34;	Zeng et al. [61]
*Camelia sinesis*	UAE	Epigallocatechin gallate	Maltose; Sorbitol; Xylitol; Maltitol;	2.41; 2.85; 2.89; 2.9;	Jeong et al. [22]
**Amide-based systems**					
*Camelia sinesis*	UAE	Epigallocatechin gallate	Urea	1.49	Jeong et al. [22]
*Nobis tangerine*	UAE	Hespiridin	Urea; Methyl urea; Acetamide;	5.10; 6.20; 6.39;	Xu et al. [21]
Buckwheat Sprouts	UAE	Isoorientin	Urea; Acetamide;	1.29; 1.57;	Mansur et al. [53]
		Isovitexin	Urea; Acetamide;	1.48; 1.83;	
*Lycium barbarum*	UAE	Morin	Urea	1.09	Ali et al. [59]
		Myricetin	Urea	2.00	
Platycladi Cacumen	UAE	Myricitrin	Methyl urea; Dimethylurea;	1.29; 1.32;	Zhuang et al. [60]
Buckwheat Sprouts	UAE	Orientin	Urea; Acetamide;	1.09; 1.22;	Mansur et al. [53]
Wild Rice Powder	UAE	Procyanidin B1	Urea	0.93	Zeng et al. [61]
		Quercetin	Urea	1.00	
Buckwheat Sprouts	UAE	Quercetin-3-*O*-robinoside	Acetamide; Urea;	1.09; 1.08;	Mansur et al. [53]
*Platycladi cacumen*	UAE	Quercitrin	Dimethylurea; Methyl urea;	1.44; 1.50;	Zhuang et al. [60]
*Lycium barbarum*; Buckwheat Sprouts	UAE	Rutin	Urea; Urea; Acetamide;	1.00; 1.23; 1.24;	Ali et al. [59]; Mansur et al. [53]
Spent Coffee	UAE	Total Flavonoids	Urea; Acetamide;	0.81; 1.11;	Yoo et al. [14]
Buckwheat Sprouts	UAE	Vitexin	Urea; Acetamide;	1.26; 1.54;	Mansur et al. [53]
**Amino-acid-based systems**					
*Camelia sinesis*	UAE	Epigallocatechin gallate	Glycine	3.10	Jeong et al. [22]
Olive Pomace	HAE	Luteolin	Glycine	11.00	Chanioti et al. [15]
		Rutin	Glycine	8.80	
**Organic-acid-based systems**					
Virgin Olive Oil	H/S	Apigenin	Lactic Acid ^a^; Propanedioic Acid ^a^;	30.33; 34.33;	Garcia et al. [16]
*Camelia sinesis*	UAE	Epigallocatechin gallate	Citric Acid	2.79	Jeong et al. [22]
*Nobis tangerine*	UAE	Hespiridin	Tartaric Acid; Citric Acid; Malic Acid; Propanedioic Acid; Levulinic Acid; Oxalic Acid; Lactic Acid;	4.35; 4.85; 5.15; 5.55; 5.75; 5.75; 5.8;	Xu et al. [21]
Buckwheat Sprouts	UAE	Isoorientin	Oxalic Acid; Propanedioic Acid;	0.92; 1.31;	Mansur et al. [53]
		Isovitexin	Oxalic Acid; Propanedioic Acid;	1.06; 1.44;	
Virgin Olive Oil; Olive Pomace	H/S; HAE	Luteolin	Lactic Acid; Propanedioic Acid; Citric Acid;	6.88; 7.50; 12.00;	Garcia et al. [16]; Chanioti et al. [15];
*Lycium barbarum*	UAE	Morin	Propanedioic Acid; Levulinic Acid; Oxalic Acid; Malic Acid; p-Toluenesulfonic Acid;	1.86; 3.27; 3.55; 3.59; 5.77;	Ali et al. [59]
		Myricetin	Oxalic Acid; Malic Acid; Propanedioic Acid; Levulinic Acid; p-Toluenesulfonic Acid;	2.25; 2.50; 3.50; 20.00; 143.00;	
*Platycladi cacumen*	UAE	Myricitrin	Levulinic Acid ^a^; Levulinic Acid ^a^;	1.42; 1.51;	Zhuang et al. [60]
Buckwheat Sprouts	UAE	Orientin	Oxalic Acid; Propanedioic Acid;	0.82; 1.09;	Mansur et al. [53]
Wild Rice Powder	UAE	Procyanidin B1	Malic Acid; Lactic Acid;	1.18; 1.35;	Zeng et al. [61]
		Quercetin	Lactic Acid	1.01	
*Juglans regia* L.	H/S	Quercetin-3-*O*-glucoside	3-Phenylpropionic Acid; 5-Phenylvaleric Acid; Citric Acid; 4-Phenylbutyric Acid; Malic Acid; Glutaric Acid; Lactic Acid; Glycolic Acid; Propanedioic Acid; Valeric Acid; Acetic Acid; Propionic Acid; Butyric Acid; Phenylacetic Acid; 3-Phenylpropionic Acid;	4.85; 5.00; 5.00; 6.38; 6.62; 7.46; 7.62; 7.77; 8.08; 8.46; 10.08; 10.62; 11.08; 11.31; 11.85;	Vieira et al. [57]
Buckwheat Sprouts	UAE	Quercetin-3-*O*-robinoside	Oxalic Acid; Propanedioic Acid;	0.41; 1.00;	Mansur et al. [53]
*Juglans regia* L.	H/S	Quercetin-*O*-pentoside	Citric Acid; 5-Phenylvaleric Acid; Propanedioic Acid; 3-Phenylpropionic Acid; Malic Acid; Glycolic Acid; 4-Phenylbutyric Acid; Lactic Acid; Glutaric Acid; Valeric Acid; Acetic Acid; Phenylacetic Acid; Propionic Acid; Butyric Acid; 3-Phenylpropionic Acid;	1.95; 2.48; 2.52; 2.62; 2.67; 3; 3.19; 3.24; 3.33; 4.52; 4.9; 5.52; 5.57; 5.71; 6.38;	Vieira et al. [57]
*Platycladi cacumen*	UAE	Quercitrin	Levulinic Acid; Levulinic Acid;	1.51; 1.60;	Zhuang et al. [60]
*Lycium barbarum;* Buckwheat Sprouts; Olive Pomace;	UAE; HAE	Rutin	Oxalic Acid; Oxalic Acid; Levulinic Acid; Propanedioic Acid; Malic Acid; Propanedioic Acid; p-Toluenesulfonic Acid; Citric Acid;	0.70; 0.72; 1.10; 1.15; 1.50; 1.80; 9.10; 17.10;	Ali et al. [59]
Spent Coffee; Fennel; Mint; Dittany; Marjoram; Sage;	UAE	Total Flavonoids	Lactic Acid ^c^; Lactic Acid ^c^; Lactic Acid ^c^; Lactic Acid ^c^; Citric Acid; Propanedioic Acid; Lactic Acid ^c^;	0.63; 0.79; 0.89; 0.90; 1.36; 1.36; 1.85;	Bakirtzi et al. [62]; Yoo et al. [14]*.;*
Buckwheat Sprouts	UAE	Vitexin	Oxalic Acid; Propanedioic Acid;	0.91; 1.22;	Mansur et al. [53]
**Sugar-based systems**					
Virgin Olive Oil	H/S	Apigenin	Sucrose ^b^; Sucrose ^b^;	19.33; 30.00;	Garcia et al. [16]
		Luteolin	Sucrose ^a^; Sucrose ^a^;	6.75; 8.13;	
*Platycladi cacumen*	UAE	Myricitrin	Glucose ^a^; Glucose ^a^;	1.08; 1.21;	Zhuang et al. [60].
		Quercitrin	Glucose ^a^; Glucose ^a^;	1.09; 1.24;	
*Nobis tangerine*	UAE	Hespiridin	Glucose; Fructose;	4.70; 4.90;	Xu et al. [21]
Wild Rice Powder	UAE	Procyanidin B1	Fructose; Glucose;	1.11; 1.71;	Zeng et al. [61]
Spent Coffee	UAE	Total Flavonoids	Fructose; Glucose; Xylose; Sucrose;	0.51; 0.58; 0.61; 0.64;	Yoo et al. [14]
Wild Rice Powder	UAE	Quercetin	Fructose; Glucose;	1.01; 1.01;	Zeng et al. [61]
*Camelia sinesis*	UAE	Epigallocatechin gallate	Sucrose; Glucose;	2.66; 2.95;	Jeong et al. [22]

^a^ betaine-based DES; ^b^ represents a different HBD/HBA ratio; ^c^ represents different natural matrices.

**Table 2:** Correlation between the natural matrix, extraction method, and DES based on ammonium salts (choline chloride and betaine) and the effect of different HBDs on the extraction efficiency of different flavonoids compared with ethanol extractions.

Natural Matrix	Extraction Method	Extracted Compound	Hydrogen Bond Donor	Ethanol EE	Reference
**Alcohol-based systems**					
Wild Rice Powder	UAE	Catechin	Glycerol; 1,4-Butanediol;	1.22; 2.23;	Zeng et al. [61]
*Hibiscus sabdariffa* L.	MAE	Cyanidin-3-sambubioside	Maltose; Ethylene Glycol; 1,2-Propanediol;	0.78; 0.83; 0.95;	Alañón et al. [63]
Delphinidin-3-sambubioside	Maltose; Ethylene Glycol; 1,2-Propanediol;	0.79; 0.81; 0.94;
*Camelia sinesis*	UAE	Epigallocatechin gallate	Maltose; Sorbitol; Xylitol; Maltitol;	0.81; 0.96; 0.97; 0.98;	Jeong et al. [25]
*Nobis tangerine*	UAE	Hespiridin	Xylitol; Maltose; 1,2-Propanediol; Glycerol; Sorbitol; Ethylene Glycol;	1.2; 1.23; 1.25; 1.3; 1.31; 1.44;	Xu et al. [21]
Buckwheat Sprouts	UAE	Isoorientin	1,2-Propanediol; Glycerol; 1,4-Butanediol; Ethylene Glycol; Triethylene Glycol;	0.50; 0.51; 0.63; 0.69; 0.95;	Mansur et al. [53]
Sea Buckthorn Leaves	MAE	Isoquercetin	1,4-Butanediol	2.08	Cui et al. [64]
		Isorhamnetin	1,4-Butanediol	2.47	
Buckwheat Sprouts	UAE	Isovitexin	Glycerol; 1,2-Propanediol; Ethylene Glycol; 1,4-Butanediol; Triethylene Glycol;	0.51; 0.60; 0.68; 0.74; 0.99;	Mansur et al. [53]
*Hibiscus sabdariffa* L.; Sea Buckthorn Leaves	MAE	Kaempferol	Maltose; Ethylene Glycol; 1,2-Propanediol; 1,4-Butanediol;	0.91; 1.09; 1.18; 1.70	Alañón et al. [63]; Cui et al. [64]
*Hibiscus sabdariffa* L.	MAE	Kaempferol-3-*O*-sambubioside	1,2-Propanediol; Maltose; Ethylene Glycol;	0.40; 0.60; 0.80;	Alañón et al. [63]
		Methylepigallocatechin	Maltose; Ethylene Glycol; 1,2-Propanediol;	0.91; 1.00; 1.18;	
*Lycium barbarum*	UAE	Morin	Glycerol; 1,2-Propanediol; Xylitol; Ethylene Glycol;	0.80; 1.2; 1.35; 3;	Ali et al. [59]
*Lycium barbarum; Hibiscus sabdariffa* L.;	UAE; MAE	Myricetin	Ethylene Glycol ^c^; Maltose; 1,2-Propanediol; Glycerol; Ethylene Glycol ^c^; 1,2-Propanediol;	0.80; 0.85; 1; 1; 1; 1.03;	Ali et al. [59]; Alañón et al. [63];
*Hibiscus sabdariffa* L.	MAE	Myricetin-3-arabinogalactoside	Maltose; Ethylene Glycol; 1,2-Propanediol;	0.93; 0.98; 1.05;	Alañón et al. [63]
Buckwheat Sprouts	UAE	Orientin	1,2-Propanediol; Glycerol; 1,4-Butanediol;	0.44; 0.50; 0.6;	Mansur et al. [53]
Wild Rice Powder	UAE	Procyanidin B1	1,4-Butanediol; Glycerol;	0.84; 0.98;	Zeng et al. [61]
*Hibiscus sabdariffa* L.; Wild Rice Powder; Sea Buckthorn Leaves;	MAE; UAE	Quercetin	Maltose; Ethylene Glycol; 1,2-Propanediol; Glycerol; 1,4-Butanediol ^c^; 1,4-Butanediol ^c^;	0.81; 0.96; 1.09; 1.25; 1.46; 3.07;	Alañón et al. [63]; Zeng et al. [61]; Cui et al. [64];
*Hibiscus sabdariffa* L.	MAE	Quercetin-3-glucoside	Maltose; 1,2-Propanediol; Ethylene Glycol;	0.82; 0.96; 1.00;	Alañón et al. [63]
Buckwheat Sprouts	UAE	Quercetin-3-*O*-robinoside	Glycerol; 1,4-Butanediol; 1,2-Propanediol; Ethylene Glycol; Triethylene Glycol;	0.51; 0.62; 0.65; 0.73; 1.09;	Mansur *et al.*
*Hibiscus sabdariffa* L.	MAE	Quercetin-3-rutinoside	Ethylene Glycol; 1,2-Propanediol; Maltose;	0.94; 0.99; 0.71;	Alañón et al. [63]
		Quercetin-3-sambubioside	Maltose; Ethylene Glycol; 1,2-Propanediol;	0.64; 0.85; 0.85;	
Buckwheat Sprouts; *Lycium* *barbarum*; Sea Buckthorn Leaves;	UAE; MAE	Rutin	Glycerol ^b,c^; Xylitol; Ethylene Glycol ^c^; 1,4-Butanediol; 1,2-Propanediol; Triethylene Glycol; Glycerol ^b,c^; 1,2-Propanediol; Ethylene Glycol ^c^; 1,4-Butanediol;	0.49; 0.71; 0.71; 0.73; 0.80; 0.96; 1.14; 1.43; 1.43; 2.05;	Mansur et al. [53]; Ali et al. [59]; Cui et al. [64];
Spent Coffee	UAE	Total Flavonoids	Sorbitol; Glycerol; Ethylene Glycol; 1,4-Butanediol; 1,6-Hexanediol;	0.34; 0.76; 0.92; 1.14; 1.26;	Yoo et al. [14]
Buckwheat Sprouts	UAE	Vitexin	Glycerol; 1,2-Propanediol; Ethylene Glycol; 1,4-Butanediol; Triethylene Glycol;	0.49; 0.57; 0.67; 0.73; 1.03;	Mansur et al. [53]
**Amide-based systems**					
*Hibiscus sabdariffa* L.	MAE	Cyanidin-3-sambubioside	Urea	0.28	Alañón et al. [63]
Delphinidin-3-sambubioside	Urea	0.38
*Camelia sinesis*	UAE	Epigallocatechin gallate	Urea; Urea;	0.50; 1.04;	Jeong et al. [25]
*Nobis tangerine*	UAE	Hespiridin	Urea; Methyl urea; Acetamide;	1.20; 1.45; 1.50;	Xu et al. [21]
Buckwheat Sprouts	UAE	Isoorientin	Urea; Acetamide;	0.73; 0.89;	Mansur et al. [53]
		Isovitexin	Urea; Acetamide;	0.75; 0.93;	
*Hibiscus sabdariffa* L.	MAE	Kaempferol	Urea	0.73	Alañón et al. [63]
		Kaempferol-3-*O*-sambubioside	Urea	1.20	
		Methylepigallocatechin	Urea	1.09	
*Lycium barbarum*	UAE	Morin	Urea	1.20	Ali et al. [59]
*Hibiscus sabdariffa L.; Lycium barbarum;*	MAE	Myricetin	Urea ^c^; Urea ^c^;	0.67; 1.6;	Alañón et al. [63]; Ali et al. [59];
*Hibiscus sabdariffa* L.	MAE	Myricetin-3-arabinogalactoside	Urea	1.45	Alañón et al. [63]
Buckwheat Sprouts	UAE	Orientin	Urea; Acetamide;	0.75; 0.84;	Mansur et al. [53]
Wild Rice Powder	UAE	Procyanidin B1	Urea	0.59	Zeng et al. [61]
*Hibiscus sabdariffa* L.; Wild Rice Powder;	MAE	Quercetin	Urea; Urea;	0.60; 1.09;	Alañón et al. [63]; Zeng et al. [61];
*Hibiscus sabdariffa* L.	MAE	Quercetin-3-glucoside	Urea	0.87	Alañón et al. [63]
Buckwheat Sprouts	UAE	Quercetin-3-*O*-robinoside	Acetamide; Urea;	0.82; 0.81;	Mansur et al. [53]
*Hibiscus sabdariffa* L.	MAE	Quercetin-3-rutinoside	Urea	0.83	Alañón et al. [63]
		Quercetin-3-sambubioside	Urea	0.90	
Buckwheat Sprouts; *Lycium* *barbarum*;	UAE	Rutin	Urea; Acetamide; Urea;	0.79; 0.80; 1.43;	Mansur et al. [53]; Ali et al. [59];
Spent Coffee	UAE	Total Flavonoids	Urea; Acetamide;	0.65; 0.89;	Yoo et al. [14]
Buckwheat Sprouts	UAE	Vitexin	Urea; Acetamide;	0.74; 0.91;	Mansur et al. [53]
**Amino-acid** **-** **based systems**					
*Camelia sinesis*	UAE	Epigallocatechin gallate	Glycine	1.04	Jeong et al. [25]
Olive Pomace	HAE	Luteolin	Glycine	1.83	Chanioti et al. [15]
		Rutin	Glycine	2.44	
**Organic** **-acid** **-** **based systems**					
*Aegle marmelos*	UAE	Apigenin	Oxalic Acid; Oxalic Acid; Oxalic Acid;	0.58; 1.58; 1.73;	Saha et al. [65]
Wild Rice Powder	UAE	Catechin	Malic Acid; Lactic Acid;	1.23; 1.52;	Zeng et al. [61]
Grape Pomace	UMAE	cyanidin -3-(6-*O*-*p*- coumaroyl)monoglucosides	Citric Acid; Malic Acid; Malic Acid; Citric Acid;	0.54; 0.67; 0.67; 0.85;	Panić et al. [66]
*Hibiscus sabdariffa* L.	MAE	Cyanidin-3-sambubioside	Lactic Acid; Oxalic Acid;	1.33; 2.08;	Alañón et al. [63]
Grape Pomace	UMAE	delphinidin-3-*O*-monoglucoside	Citric Acid ^a^; Malic Acid ^a^; Malic Acid ^a^; Citric Acid ^a^;	0.98; 1.19; 1.23; 1.48;	Panić et al. [66]
*Hibiscus sabdariffa* L.	MAE	Delphinidin-3-sambubioside	Lactic Acid; Oxalic Acid;	1.01; 1.13;	Alañón *et al.* [63]
*Camelia sinesis*	UAE	Epigallocatechin gallate	Citric Acid	0.94	Jeong et al. [25]
Nobis tangerine	UAE	Hespiridin	Tartaric Acid; Citric Acid; Malic Acid; Propanedioic Acid; Levulinic Acid; Oxalic Acid; Lactic Acid;	1.02; 1.14; 1.21; 1.3; 1.35; 1.35; 1.36;	Xu et al. [21]
Buckwheat Sprouts	UAE	Isoorientin	Oxalic Acid; Propanedioic Acid;	0.52; 0.74;	Mansur et al. [53]
		Isovitexin	Oxalic Acid; Propanedioic Acid;	0.54; 0.73;	
*Aegle marmelos*	UAE	Kaempferol	Oxalic Acid; Lactic Acid; Oxalic Acid; Oxalic Acid; Oxalic Acid;	0.52; 1.09; 1.53; 1.65; 2.45;	Saha et al. [65]
*Hibiscus sabdariffa* L.	MAE	Kaempferol-3-*O*-sambubioside	Lactic Acid; Oxalic Acid;	0.60; 2.20;	Alañón et al. [63]
Olive Pomace	HAE	Luteolin	Citric Acid	2.00	Chanioti et al. [15]
Grape Pomace	UMAE	malvidin-3-(6-*O*-*p*-coumaroyl)monoglucosides	Citric Acid ^a^; Malic Acid ^a^; Malic Acid ^a^; Citric Acid ^a^;	0.53; 0.54; 0.71; 0.87;	Panić et al. [66]
		malvidin-3-*O*-acetylmonoglucoside	Citric Acid ^a^; Malic Acid ^a^; Malic Acid ^a^; Citric Acid ^a^;	0.63; 0.79; 0.89; 1.04;	
		malvidin-3-*O*-monoglucoside	Citric Acid ^a^; Malic Acid ^a^; Malic Acid ^a^; Citric Acid ^a;^	0.8; 0.84; 1.05; 1.26;	
*Hibiscus sabdariffa* L.	MAE	Methylepigallocatechin	Oxalic Acid; Lactic Acid;	0.73; 1.00;	Alañón et al. [63]
*Lycium barbarum*	UAE	Morin	Propanedioic Acid; Levulinic Acid; Oxalic Acid; Malic Acid; *p*-Toluenesulfonic Acid;	2.05; 3.6; 3.9; 3.95; 6.35;	Ali et al. [59]
*Hibiscus sabdariffa L.; Lycium barbarum;*	MAE; UAE	Myricetin	Lactic Acid; Oxalic Acid ^b,c^; Oxalic Acid ^b,c^; Malic Acid; Propanedioic Acid; Levulinic Acid; *p*-Toluenesulfonic Acid;	1.10; 1.46; 1.8; 2; 2.8; 16; 114.4;	Alañón et al. [63]; Ali et al. [59];
*Hibiscus sabdariffa* L.	MAE	Myricetin-3-arabinogalactoside	Lactic Acid; Oxalic Acid;	0.86; 2.62;	Alañón et al. [63]
Buckwheat Sprouts	UAE	Orientin	Oxalic Acid; Propanedioic Acid;	0.56; 0.75;	Mansur et al. [53]
Grape Pomace	UMAE	peonidin-3-(6-*O*-*p*-coumaroyl)monoglucosides	Citric Acid ^a^; Malic Acid ^a^; Malic Aci ^a^d; Citric Acid ^a^;	0.58; 0.72; 0.72; 0.91;	Panić et al. [66]
		peonidin-3-*O*-acetylmonoglucoside	Citric Acid ^a^; Malic Acid ^a^; Malic Aci ^a^d; Citric Acid ^a^;	0.65; 0.79; 0.81; 0.99;	
		peonidin-3-*O*-monoglucoside	Citric Acid ^a^; Malic Acid ^a^; Malic Aci ^a^d; Citric Acid ^a^;	0.73; 0.85; 0.97; 1.06;	
		petunidin-3-*O*-monoglucoside	Citric Acid ^a^; Malic Acid ^a^; Malic Aci ^a^d; Citric Acid ^a^;	1; 1.21; 1.25; 1.51;	
Wild Rice Powder	UAE	Procyanidin B1	Malic Acid; Lactic Acid;	0.75; 0.85;	Zeng et al. [61]
*Hibiscus sabdariffa* L.; Wild Rice Powder;	MAE; UAE	Quercetin	Lactic Acid ^c^; Lactic Acid ^c^; Oxalic Acid;	1.03; 1.09; 1.89;	Alañón et al. [63]; Zeng et al. [61];
*Hibiscus sabdariffa* L.	MAE	Quercetin-3-glucoside	Oxalic Acid; Lactic Acid;	0.55; 1.00;	Alañón et al. [63]
*Juglans regia* L.	H/S	Quercetin-3-*O*-glucoside	3-Phenylpropionic Acid; 5-Phenylvaleric Acid; Citric Acid; 4-Phenylbutyric Acid; Malic Acid; Glutaric Acid; Lactic Acid; Glycolic Acid; Propanedioic Acid; Valeric Acid; Acetic Acid; Propionic Acid; Butyric Acid; Phenylacetic Acid; 3-Phenylpropionic Acid;	0.56; 0.58; 0.58; 0.73; 0.76; 0.86; 0.88; 0.89; 0.93; 0.97; 1.16; 1.22; 1.27; 1.30; 1.36;	Vieira et al. [57]
Buckwheat Sprouts	UAE	Quercetin-3-*O*-robinoside	Oxalic Acid; Propanedioic Acid;	0.30; 0.75;	Mansur et al. [53]
*Hibiscus sabdariffa* L.	MAE	Quercetin-3-rutinoside	Oxalic Acid; Lactic Acid;	0.23; 0.91;	Alañón et al. [63]
		Quercetin-3-sambubioside	Oxalic Acid; Lactic Acid;	0.38; 0.71;	
*Juglans regia* L.	H/S	Quercetin-O-pentoside	Citric Acid; 5-Phenylvaleric Acid; Propanedioic Acid; 3-Phenylpropionic Acid; Malic Acid; Glycolic Acid; 4-Phenylbutyric Acid; Lactic Acid; Glutaric Acid; Valeric Acid; Acetic Acid; Phenylacetic Acid; Propionic Acid; Butyric Acid; 3-Phenylpropionic Acid;	0.48; 0.6; 0.62; 0.64; 0.65; 0.73; 0.78; 0.79; 0.81; 1.10; 1.20; 1.35; 1.36; 1.40; 1.56;	Vieira et al. [57]
Buckwheat Sprouts	UAE	Rutin	Oxalic Acidc; Propanedioic Acidc; Oxalic Acidc; Levulinic Acid; Malic Acid; Propanedioic Acidc; p-Toluenesulfonic Acid;	0.51; 0.82; 0.88; 1.38; 1.88; 2.25; 11.38;	Mansur et al. [53]
Spent Coffee; Fennel; Mint; Dittany; Marjoram; Sage;	UAE	Total Flavonoids	Lactic Acidc ^c^; Lactic Acid ^c^; Lactic Acid ^c^; Lactic Acid; Citric Acid; Propanedioic Acid; Lactic Acid ^c^;	0.62; 0.66; 0.88; 0.88; 1.08; 1.09; 1.09;	Bakirtzi et al. [62]; Yoo et al. [14];
Buckwheat Sprouts	UAE	Vitexin	Oxalic Acid; Propanedioic Acid;	0.54; 0.72;	Mansur et al. [53]
**Sugar-based systems**					
Wild Rice Powder	UAE	Catechin	Glucose; Fructose;	0.97; 1.11;	Zeng et al. [61]
*Hibiscus sabdariffa* L.	MAE	Cyanidin-3-sambubioside	Fructose; Glucose;	0.82; 0.87;	Alañón et al. [63]
Delphinidin-3-sambubioside	Fructose; Glucose;	0.83; 0.86;
*Camelia sinesis*	UAE	Epigallocatechin gallate	Sucrose; Glucose;	0.90; 0.99;	Jeong et al. [25]
*Nobis tangerine*	UAE	Hespiridin	Glucose; Fructose;	1.10; 1.15;	Xu et al. [21]
*Hibiscus sabdariffa* L.	MAE	Kaempferol	Fructose; Glucose;	0.91; 0.91;	Alañón et al. [63]
		Kaempferol-3-*O*-sambubioside	Fructose; Glucose;	0.60; 0.80;	
		Methylepigallocatechin	Glucose; Fructose;	0.91; 1.00;	
		Myricetin	Fructose; Glucose;	0.89; 0.93;	
		Myricetin-3-arabinogalactoside	Fructose; Glucose;	0.90; 0.95;	
		Neochlorogenic acid	Fructose; Glucose;	0.83; 0.84;	
Wild Rice Powder	UAE	Procyanidin B1	Fructose; Glucose;	0.70; 1.08;	Zeng et al. [61]
*Hibiscus sabdariffa* L.; Wild Rice Powder;	MAE	Quercetin	Glucose; Fructose; Fructose; Glucose;	0.83; 0.85; 1.09; 1.10;	Alañón et al. [63]; Zeng et al. [61];
*Hibiscus sabdariffa* L.	MAE	Quercetin-3-glucoside	Fructose; Glucose;	0.85; 0.89;	Alañón et al. [63]
		Quercetin-3-rutinoside	Fructose; Glucose;	0.77; 0.80;	
		Quercetin-3-sambubioside	Glucose; Fructose;	0.69; 0.71;	
Spent Coffee	UAE	Total Flavonoids	Fructose; Glucose; Xylose; Sucrose;	0.41; 0.47; 0.49; 0.51;	Yoo et al. [14]

^a^ betaine-based DES; ^b^ represents a different HBD/HBA ratio; ^c^ represents different natural matrices.

**Table 3:** Correlation between the natural matrix, extraction method, and DES based on ammonium salts (choline chloride and betaine) and the effect of different HBDs on the extraction efficiency of different flavonoids compared with methanol extractions.

Natural Matrix	Extraction Method	Extracted Compound	Hydrogen Bond Donor	Methanol EE	Reference
**Alcohol-based systems**					
*Platycladi cacumen*	UAE	Amentoflavone	Ethylene Glycol ^a^; Ethylene Glycol ^a^;	1.04; 1.15;	Zhuang et al. [60]
*Camelia sinesis* Seed Oil; Virgin Olive Oil;	H/S	Apigenin	Propilene Glycol; 1,4-Butanediol; Glycerol; Xylitol; Glycerol; Xylitol; 1,2-Propanediol;	0.45; 0.92; 0.92; 0.93; 0.96; 1.00; 1.15;	Garcia et al. [16]; Wang et al. [67];
*Camelia sinesis* Seed Oil	H/S	Catechin	Glycerol; Propilene Glycol; Ethylene Glycol; Xylitol; Glycerol;	0.06; 0.06; 0.12; 0.16; 1.00;	Wang et al. [67]
Grape Skin	UAE	Cyanidin-3-*O*-monoglucoside	Glycerol	0.67	Radošević et al. [17]
Grape Skin	UAE	Delphinidin-3-*O*-monoglucoside	Glycerol	1.00	Radošević et al. [17]
*Camelia sinesis* Seed Oil	H/S	Epicatechin	Ethylene Glycol; Glycerol; Propilene Glycol; Xylitol;	0.02; 0.03; 0.13; 0.61;	Wang et al. [67]
		Epicatechin gallate	Glycerol; Xylitol; Ethylene Glycol; Propilene Glycol;	1.00; 1.00; 1.00; 1.00;	
		Epigallocatechin	Ethylene Glycol; Propilene Glycol; Xylitol;	1.01; 1.01; 1.02;	
*Camelia sinesis*	UAE	Epigallocatechin gallate	Maltitol ^a^; Xylitol ^a^; Maltose ^a^; Sorbitol ^a^; Sorbitol ^a^; Maltose ^a^; Xylitol ^a^; Sorbitol ^a^; Xylitol ^a^; Maltose ^a^; Maltitol ^a^; Maltitol ^a^;	0.73; 0.77; 0.77; 0.78; 0.78; 0.85; 0.91; 1.01; 1.02; 1.03; 1.03; 1.06;	Jeong et al. [22]
*Nobis tangerine*	UAE	Hespiridin	Xylitol; Maltose; 1,2-Propanediol; Glycerol; Sorbitol; Ethylene Glycol;	0.95; 0.97; 0.99; 1.03; 1.04; 1.14;	Xu et al. [21]
*Platycladi cacumen*	UAE	Hinokiflavone	Ethylene Glycol ^a^; Ethylene Glycol ^a^;	0.59; 0.96;	Zhuang et al. [60].
Buckwheat Sprouts	UAE	Isoorientin	1,2-Propanediol; Glycerol; 1,4-Butanediol; Ethylene Glycol; Triethylene Glycol;	0.57; 0.58; 0.72; 0.78; 1.08;	Mansur et al. [53]
		Isovitexin	Glycerol; 1,2-Propanediol; Ethylene Glycol; 1,4-Butanediol; Triethylene Glycol;	0.57; 0.66; 0.76; 0.82; 1.11;	
*Camelia sinesis* Seed Oil	H/S	Kaempferol	Propilene Glycol; Ethylene Glycol; Glycerol;	0.49; 0.90; 1.12;	Wang et al. [67]
*Camelia sinesis* Seed Oil; Lycium Barbarum; Virgin Olive Oil;	H/S; UAE; H/S;	Luteolin	Ethylene Glycol ^c^; Propilene Glycol; Ethylene Glycol ^c^; 1,4-Butanediol; Glycerol; Xylitol; 1,2-Propanediol ^c^; Glycerol; 1,2-Propanediol ^c^;	0.43; 0.46; 0.49; 1.00; 1.00; 1.09; 1.19; 1.2; 1.49;	Wang et al. [67] Ali et al. [59]; Garcia et al. [16];
Grape Skin	UAE	Malvidin-3-(6-*O*-*p*-coumaroyl)monoglucoside	Glycerol	1.60	Radošević et al. [17]
		Malvidin-3-*O*-acetylmonoglucoside	Glycerol	2.00	
		Malvidin-3-*O*-monoglucoside	Glycerol	0.81	
*Lycium barbarum*	UAE	Morin	Glycerol; 1,2-Propanediol; Xylitol; Ethylene Glycol;	0.67; 1.00; 1.13; 2.50;	Ali et al. [59]
		Myricetin	Ethylene Glycol; 1,2-Propanediol; Glycerol;	1.00; 1.25; 1.25;	
*Platycladi cacumen*	UAE	Myricitrin	Glycerol; Ethylene Glycol; Ethylene Glycol;	1.28; 1.40; 1.41;	Zhuang et al. [60]
*Camelia sinesis* Seed Oil	H/S	Naringenin	Ethylene Glycol; Glycerol; Propilene Glycol; Xylitol;	0.37; 0.40; 0.48; 0.93;	Wang et al. [67]
Flos Sophorae	UAE	Nicotiflorin	Xylitol	0.91	Nam et al. [25]
Buckwheat Sprouts	UAE	Orientin	1,2-Propanediol; Glycerol; 1,4-Butanediol; Ethylene Glycol; Triethylene Glycol;	0.50; 0.58; 0.68; 0.81; 1.12;	Mansur et al. [53]
Grape Skin	UAE	Peonidin-3-(6-*O*-*p*-coumaroyl)monoglucoside	Glycerol	1.00	Radošević et al. [17]
		Peonidin-3-*O*-monoglucoside	Glycerol	1.17	
		Petunidin-3-*O*-monoglucoside	Glycerol	1.00	
		Quercetin-3-*O*-glucoside	Glycerol	1.00	
Buckwheat Sprouts	UAE	Quercetin-3-*O*-robinoside	Glycerol; 1,4-Butanediol; 1,2-Propanediol; Ethylene Glycol; Triethylene Glycol;	0.55; 0.67; 0.70; 0.79; 1.19;	Mansur et al. [53]
*Platycladi cacumen*	UAE	Quercitrin	Glycerol; Ethylene Glycol ^b^; Ethylene Glycol ^b^;	1.27; 1.38; 1.40;	Zhuang et al. [60]
		Quercitrin	Levulinic Acid	1.45	
Tartary buckwheat hull; Buckwheat Sprouts; *Lycium barbarum;*	UAE	Rutin	Sorbitol; Glycerol ^c^; Xylitol ^c^; Xylitol ^c^; Ethylene Glycol ^c^; 1,4-Butanediol; 1,2-Propanediol; Glycerol ^c^; Triethylene Glycol; 1,2-Propanediol; Ethylene Glycol ^c^; 1,2-Propanediol; Glycerol ^c^;	0.45; 0.53; 0.63; 0.66; 0.77; 0.8; 0.88; 1.00; 1.05; 1.25; 1.25; 1.48; 1.98;	Huang et al. [68]; Mansur et al. [53]; Ali et al. [59];
*Camelia sinesis* Seed Oil	H/S	Taxifolin	Ethylene Glycol; Xylitol; Glycerol;	0.44; 2.39; 3.95;	Wang et al. [67]
Spent Coffee; *Lippia citriodora*	UAE; MAE	Total Flavonoids	Sorbitol; Glycerol; Ethylene Glycol ^c^; Xylitol; 1,3-Butanediol; Maltose; 1,4-Butanediol; 1,6-Hexanediol; 1,2-Propanediol; Ethylene Glycol ^c^;	0.34; 0.77; 0.93; 1.00; 1.02; 1.06; 1.14; 1.27; 1.38; 1.42;	Yoo et al. [14].; Ivanović et al. [63]
*Camelia sinesis* Seed Oil; Buckwheat Sprouts	H/S; UAE	Vitexin	Glycerolc; Propilene Glycol; Glycerolc; Ethylene Glycolc; 1,2-Propanediol; Ethylene Glycolc; 1,4-Butanediol; Triethylene Glycol; Xylitol;	0.41; 0.52; 0.56; 0.64; 0.66; 0.77; 0.84; 1.19; 1.52;	Wang et al. [67]; Mansur et al. [53]
**Amide-based systems**					
*Platycladi cacumen*	UAE	Amentoflavone	Methyl urea; Dimethylurea;	0.92; 1.12;	Zhuang et al. [60]
*Camelia sinesis*	UAE	Epigallocatechin gallate	Urea	0.53	Jeong et al. [22]
*Nobis tangerine*	UAE	Hespiridin	Urea; Methyl urea; Acetamide;	0.95; 1.15; 1.19;	Xu et al. [21]
*Platycladi cacumen*	UAE	Hinokiflavone	Methyl urea; Dimethylurea;	0.47; 0.94;	Zhuang et al. [60]
Buckwheat Sprouts	UAE	Isoorientin	Urea; Acetamide;	0.83; 1;	Mansur et al. [53]
		Isovitexin	Urea; Acetamide;	0.84; 1.04;	
*Lycium barbarum*	*UAE*	Luteolin	Urea	0.12	Ali et al. [59]
		Morin	Urea	1.00	
		Myricetin	Urea	2.00	
*Platycladi cacumen*	UAE	Myricitrin	Methyl urea; Dimethylurea; Acetamide;	1.27; 1.31; 1.47;	Zhuang et al. [60]
Buckwheat Sprouts	UAE	Orientin	Urea; Acetamide;	0.86; 0.96;	Mansur et al. [53]
		Quercetin-3-*O*-robinoside	Urea; Acetamide;	0.88; 0.89;	
*Platycladi cacumen*	UAE	Quercitrin	Dimethylurea; Methyl urea; Acetamide;	1.31; 1.36; 1.40;	Zhuang et al. [60]
Buckwheat Sprouts	UAE	Rutin	Urea; Acetamide; Urea;	0.87; 0.88; 1.25;	Mansur et al. [53]
Spent Coffee	UAE	Total Flavonoids	Urea; Acetamide; Urea;	0.65; 0.89; 0.96;	Yoo et al. [14]
Buckwheat Sprouts	UAE	Vitexin	Urea; Acetamide;	0.85; 1.04;	Mansur et al. [53]
**Amino-acid-based systems**					
*Camelia sinesis*	UAE	Epigallocatechin gallate	Glycinea; Glycinea;	1.03; 1.10;	Jeong et al. [22]
Flos Sophorae	UAE	Narcissim	Glycine	1.33	Nam et al. [25]
		Nicotiflorin	Glycine	1.09	
		Rutin	Glycine	1.05	
**Organic-acid-based systems**					
*Platycladi cacumen*	UAE	Amentoflavone	Levulinic Acida; Levulinic Acida;	1.28; 1.42;	Zhuang et al. [60].
Virgin Olive Oil	H/S	Apigenin	Lactic Acid; Propanedioic Acid;	0.70; 0.79;	Garcia et al. [16]
*Camelia sinesis* Seed Oil	H/S	Catechin	Propanedioic Acid; Malic Acid;	0.04; 4.00;	Wang et al. [67]
Grape Skin	UAE	Cyanidin-3-*O*-monoglucoside	Malic Acid	1.00	Radošević et al. [17]
		Delphinidin-3-*O*-monoglucoside	Malic Acid	1.50	
*Camelia sinesis* Seed Oil	H/S	Epicatechin	Propanedioic Acid	0.13	Wang et al. [67]
		Epicatechin gallate	Propanedioic Acid	1.00	
		Epigallocatechin	Propanedioic Acid	1.00	
*Camelia sinesis*	UAE	Epigallocatechin gallate	Citric Acid	0.99	Jeong et al. [22]
*Nobis tangerine*	UAE	Hespiridin	Tartaric Acid; Citric Acid; Malic Acid; Propanedioic Acid; Levulinic Acid; Oxalic Acid; Lactic Acid;	0.81; 0.90; 0.95; 1.03; 1.07; 1.07; 1.07;	Xu et al. [21]
*Platycladi cacumen*	UAE	Hinokiflavone	Levulinic Acid; Levulinic Acid;	1.22; 1.50;	Zhuang et al. [60]
Buckwheat Sprouts	UAE	Isoorientin	Oxalic Acid; Propanedioic Acid;	0.59; 0.83;	Mansur et al. [53]
		Isovitexin	Oxalic Acid; Propanedioic Acid;	0.60; 0.82;	
*Camelia sinesis* Seed Oil	H/S	Kaempferol	Propanedioic Acid	0.51	Wang et al. [67]
*Lycium barbarum*	UAE	Luteolin	Propanedioic Acid; Malic Acid; Oxalic Acid; Propanedioic Acid; *p*-Toluenesulfonic Acid; Lactic Acid; Propanedioic Acid;	0.11; 0.19; 0.21; 0.47; 0.60; 0.81; 0.88;	Ali et al. [59]
Grape Skin	UAE	Malvidin-3-(6-*O*-*p*-coumaroyl)monoglucoside	Malic Acid	2.30	Radošević et al. [17]
		Malvidin-3-*O*-acetylmonoglucoside	Malic Acid	2.00	
		Malvidin-3-*O*-monoglucoside	Malic Acid	1.48	
*Lycium barbarum*	*UAE*	Morin	Propanedioic Acid; Levulinic Acid; Oxalic Acid; Malic Acid; *p*-Toluenesulfonic Acid;	1.71; 3.00; 3.25; 3.29; 5.29;	Ali et al. [59]
		Myricetin	Oxalic Acid; Malic Acid; Propanedioic Acid; Levulinic Acid; *p*-Toluenesulfonic Acid;	2.25; 2.50; 3.50; 20.00; 143.00;	
Platycladi Cacumen	UAE	Myricitrin	Levulinic Acid; Levulinic Acid; Levulinic Acid;	1.40; 1.49; 1.49;	Zhuang et al. [60]
*Camelia sinesis* Seed Oil	H/S	Naringenin	Propanedioic Acid	0.22	Wang et al. [67]
Buckwheat Sprouts	UAE	Orientin	Oxalic Acid; Propanedioic Acid;	0.64; 0.85;	Mansur et al. [53]
Grape Skin	UAE	Peonidin-3-(6-*O*-*p*-coumaroyl)monoglucoside	Malic Acid	1.50	Radošević et al. [17]
		Peonidin-3-*O*-monoglucoside	Malic Acid	3.17	
		Petunidin-3-*O*-monoglucoside	Malic Acid	3.00	
		Quercetin-3-*O*-glucoside	Malic Acid	0.67	
Buckwheat Sprouts	UAE	Quercetin-3-*O*-robinoside	Oxalic Acid; Propanedioic Acid;	0.33; 0.81;	Mansur et al. [53]
*Platycladi cacumen*	UAE	Quercitrin	Levulinic Acid; Levulinic Acid;	1.38; 1.45;	Zhuang et al. [60]
Buckwheat Sprouts	UAE	Rutin	Oxalic Acidc; Propanedioic Acidc; Oxalic Acidc; Levulinic Acid; Malic Acid; Propanedioic Acidc; *p*-Toluenesulfonic Acid;	0.51; 0.82; 0.88; 1.38; 1.88; 2.25; 11.38;	Mansur et al. [53]
*Camelia sinesis* Seed Oil	H/S	Taxifolin	Propanedioic Acid	0.39	Wang et al. [67]
Spent Coffee	UAE	Total Flavonoids	Citric Acid; Propanedioic Acid; Tartaric Acid; Lactic Acid;	1.09; 1.10; 1.31; 1.66;	Yoo et al. [14]
*Camelia sinesis* Seed Oil; Buckwheat Sprouts	H/S; UAE	Vitexin	Propanedioic Acid; Oxalic Acid; Propanedioic Acid;	0.24; 0.62; 0.83;	Wang et al. [67]; Mansur et al. [53]
**Sugar-based systems**					
*Platycladi cacumen*	UAE	Amentoflavone	Glucose; Glucose;	0.12; 0.17;	Zhuang et al. [60]
Virgin Olive Oil	H/S	Apigenin	Sucrose; Sucrose;	0.45; 0.69;	Garcia et al. [16]
Grape Skin	UAE	Catechin	Glucose; Fructose; Xylose;	4.00; 5.00; 6.00;	Radošević et al. [17]
		Cyanidin-3-*O*-monoglucoside	Fructose; Glucose; Xylose;	0.67; 0.67; 0.67;	
		Delphinidin-3-*O*-monoglucoside	Xylose; Fructose; Glucose;	1.13; 1.00; 0.50;	
*Camelia sinesis*	UAE	Epigallocatechin gallate	Sucrose; Glucose;	0.94; 1.05;	Jeong et al. [22]
*Nobis tangerine*	UAE	Hespiridin	Glucose; Fructose;	0.87; 0.91;	Xu et al. [21]
Virgin Olive Oil	H/S	Luteolin	Sucrose ^b^; Sucrose ^b^;	0.79; 0.96;	Garcia et al. [16]
Grape Skin	UAE	Malvidin-3-(6-*O*-*p*-coumaroyl)monoglucoside	Glucose; Fructose; Xylose;	2.20; 2.30; 2.40;	Radošević et al. [17]
Grape Skin	UAE	Malvidin-3-*O*-acetylmonoglucoside	Fructose; Glucose; Xylose;	2.00; 2.00; 2.00;	Radošević et al. [17]
Grape Skin	UAE	Malvidin-3-*O*-monoglucoside	Glucose; Fructose; Xylose;	1.24; 1.35; 1.43;	Radošević et al. [17]
*Platycladi cacumen*	UAE	Myricitrin	Glucose ^a^; Glucose ^a^;	1.06; 1.19;	Zhuang et al. [60]
Grape Skin	UAE	Peonidin-3-(6-*O*-*p*-coumaroyl)monoglucoside	Fructose; Glucose; Xylose;	1.00; 1.00; 1.00;	Radošević et al. [17]
Grape Skin	UAE	Peonidin-3-*O*-monoglucoside	Glucose; Fructose; Xylose;	1.00; 1.67; 3.00;	Radošević et al. [17]
Grape Skin	UAE	Petunidin-3-*O*-monoglucoside	Fructose; Glucose; Xylose;	2.00; 2.00; 2.00;	Radošević et al. [17]
Grape Skin	UAE	Quercetin-3-*O*-glucoside	Glucose; Xylose; Fructose;	0.33; 0.67; 1.00;	Radošević et al. [17]
*Platycladi cacumen*	UAE	Quercitrin	Glucose; Glucose;	0.99; 1.13;	Zhuang et al. [60]
Tartary buckwheat hull	UAE	Rutin	Glucose	1.12	Huang et al. [68]
Spent Coffee	UAE	Total Flavonoids	Fructose ^c^; Glucose ^c^; Xylose; Sucrose ^c^; Glucose ^c^; Fructose ^c^; Sucrose ^c^;	0.41; 0.47; 0.49; 0.52; 1.59; 1.02; 1.00;	Yoo et al. [14]

^a^ represents a difference in the HBA; ^b^ represents a different HBD/HBA ratio; ^c^ represents different natural matrices.

**Table 4:** Correlation between the natural matrix, extraction method, and DES based on ammonium salts (choline chloride and betaine) and the effect of different HBDs on the extraction efficiency of different phenolic acids compared with water extractions.

Natural Matrix	Extraction Method	Extracted Compound	Hydrogen Bond Donor	Water EE	Reference
**Alcohol-based systems**					
*Lonicerae japonicae Flos*	MAE	3,4-Dicafeoylquinic Acid	1,2-Propanediol; Sorbitol; 1,4-Butanediol; Glycerol;	15.6; 16; 19.6; 20.4;	Peng et al. [69]
		3,5-Dicafeoylquinic Acid	1,2-Propanediol; Sorbitol; Glycerol; Ethylene Glycol; 1,4-Butanediol; 1,3-Butanediol;	0.91; 0.94; 1.05; 1.14; 1.32; 1.65;	
		4,5-Dicafeoylquinic Acid	Sorbitol; 1,2-Propanediol; Glycerol; 1,4-Butanediol; 1,3-Butanediol; Ethylene Glycol;	1.18; 1.56; 1.74; 2.01; 2.38; 2.67;	
		Caffeic Acid	1,2-Propanediol; Sorbitol; 1,4-Butanediol; Glycerol; Ethylene Glycol; 1,3-Butanediol;	0.89; 1.03; 1.3; 1.51; 2.03; 2.16;	
		Chlorogenic Acid	1,2-Propanediol; Glycerol; Sorbitol; 1,4-Butanediol; Ethylene Glycol; 1,3-Butanediol;	0.95; 1.09; 1.13; 1.26; 1.26; 1.53;	
Wild Rice Powder	UAE	Ferulic acid	Glycerol; 1,4-Butanediol;	1.61; 1.95;	Zeng et al. [61]
		*p*-Coumaric acid	1,4-Butanediol	0.24	
		*p*-Hydroxybenzaldehyde	1,4-Butanediol; Glycerol;	1.31; 3.10;	
		*p*-Hydroxybenzoic acid	Glycerol; 1,4-Butanediol;	1.54; 1.72;	
		Protocatechuic acid	1,4-Butanediol; Glycerol;	0.64; 0.70;	
		Sinapic acid	Glycerol; 1,4-Butanediol;	1.63; 2.08;	
		Syringic acid	1,4-Butanediol	0.59	
Orange Peel; Spent Coffee	H/S; UAE	Total Phenolics	Ethylene Glycol ^a^; Sorbitol; Glycerol ^a^; Ethylene Glycol ^a^; Ethylene Glycol ^a^; Glycerol ^a^; Glycerol ^a^; Ethylene Glycol ^a^; Glycerol ^a^; 1,4-Butanediol; 1,6-Hexanediol;	0.82; 0.83; 0.87; 1.17; 1.44; 1.51; 1.57; 1.58; 1.84; 2.18; 2.32;	Ozturk et al. [70]; Yoo et al. [14]
Wild Rice Powder	UAE	Vanilin	1,4-Butanediol; Glycerol;	0.87; 1.04;	Zeng et al. [61]
		Vanillic acid	Glycerol; 1,4-Butanediol;	0.67; 0.91;	
**Amide-based systems**					
*Lonicerae japonicae Flos*	MAE	3,4-Dicafeoylquinic Acid	Urea	119.20	Peng et al. [69]
		3,5-Dicafeoylquinic Acid		0.43	
		4,5-Dicafeoylquinic Acid		1.99	
		Caffeic Acid		2.97	
		Chlorogenic Acid		0.60	
Wild Rice Powder	UAE	ferulic acid	Urea	1.37	Zeng et al. [61]
		*p*-Coumaric acid		2.46	
		*p*-Hydroxybenzaldehyde		2.06	
		*p*-Hydroxybenzoic acid		1.68	
		Protocatechuic acid		2.56	
		Sinapic acid		1.39	
		Syringic acid		0.64	
Spent Coffee	UAE	Total Phenolics	Urea; Acetamide;	0.91; 1.65;	Yoo et al. [14]
Wild Rice Powder	UAE	Vanilin	Urea	1.45	Zeng et al. [61]
		Vanillic acid		0.68	
**Organic-acid-based systems**					
*Lonicerae japonicae Flos*	MAE	3,4-Dicafeoylquinic Acid	Propanedioic Acid	18.40	Peng et al. [69]
		Caffeic Acid	Propanedioic Acid	9.46	
		4,5-Dicafeoylquinic Acid	Propanedioic Acid	1.67	
Wild Rice Powder	UAE	*p*-Coumaric acid	Lactic Acid; Malic Acid;	1.18; 3.36;	Zeng *et al.* [61]
		Protocatechuic acid	Malic Acid; Lactic Acid;	1.85; 2.06;	
		*p*-Hydroxybenzaldehyde	Lactic Acid; Malic Acid;	0.07; 4.06;	
*Juglans regia L*.; *Lonicerae japonicae Flos*	H/S; MAE	Chlorogenic Acid	5-Phenylvaleric Acid; 3-Phenylpropionic Acid; 4-Phenylbutyric Acid; Citric Acid; Glutaric Acid; Acetic Acid; Phenylacetic Acid; Malic Acid; 3-Phenylpropionic Acid; Valeric Acid; Propanedioic Acid; Lactic Acid; Glycolic Acid; Butyric Acid; Propionic Acid; Propanedioic Acid;	0.39; 0.46; 0.70; 0.81; 0.81; 0.91; 0.91; 0.93; 1.00; 1.00; 1.04; 1.06; 1.15; 1.35; 1.44; 1.23;	Vieira et al. [57]; Peng et al. [69]
Wild Rice Powder	UAE	*p*-Hydroxybenzoic acid	Lactic Acid; Malic Acid;	1.38; 1.74;	Zeng et al. [61]
		Syringic acid	Lactic Acid; Malic Acid;	0.20; 0.62;	
		Vanilin	Lactic Acid; Malic Acid;	1.21; 1.66;	
*Lonicerae japonicae Flos*	MAE	3,5-Dicafeoylquinic Acid	Propanedioic Acid	1.29	Peng et al. [69]
Wild Rice Powder	UAE	Vanillic acid	Malic Acid	0.57	Zeng et al. [61]
		Sinapic acid	Lactic Acid; Malic Acid;	1.38; 1.61;	
		Ferulic acid	Lactic Acid; Malic Acid;	0.99; 1.31;	
Dittany; Fennel; Sage; Marjoram; Spent Coffee; Mint;	UAE	Total Phenolics	Lactic Acid; Lactic Acid; Lactic Acid; Lactic Acid; Propanedioic Acid; Citric Acid; Lactic Acid;	1.45; 1.50; 1.56; 1.74; 1.77; 1.78; 2.13;	Bakirtzi et al. [62]; Yoo et al. [14]
**Sugar-based systems**					
*Lonicerae japonicae Flos*	MAE	3,4-Dicafeoylquinic Acid	Glucose	13.60	Peng et al. [69]
		Caffeic Acid	Glucose	0.78	
		4,5-Dicafeoylquinic Acid	Glucose	0.90	
Wild Rice Powder	UAE	*p*-Coumaric acid	Fructose; Glucose;	0.35; 0.76;	Zeng et al. [61]
		Protocatechuic acid	Fructose; Glucose;	0.98; 1.58;	
		*p*-Hydroxybenzaldehyde	Fructose; Glucose;	0.48; 1.93;	
		*p*-Hydroxybenzoic acid	Glucose; Fructose;	1.26; 1.43;	
		Syringic acid	Fructose; Glucose;	0.34; 0.39;	
		Vanilin	Fructose; Glucose;	0.76; 0.98;	
*Lonicerae japonicae Flos*	MAE	3,5-Dicafeoylquinic Acid	Glucose	0.78	Peng et al. [69]
		Chlorogenic Acid	Glucose	0.88	
Wild Rice Powder	UAE	Vanillic acid	Glucose; Fructose;	0.92; 1.02;	Zeng et al. [61]
		Sinapic acid	Glucose; Fructose;	0.84; 1.92;	
		Ferulic acid	Glucose; Fructose;	0.12; 1.83;	
Spent Coffee	UAE	Total Phenolics	Glucose; Sucrose; Xylose; Fructose;	0.49; 0.72; 1.52; 2.94;	Yoo et al. [14]

^a^ represents a different HBD/HBA ratio.

**Table 5:** Correlation between the natural matrix, extraction method, and DES based on ammonium salts (choline chloride and betaine) and the effect of different HBDs on the extraction efficiency of different phenolic acids compared with ethanol extractions.

Natural Matrix	Extraction Method	Extracted Compound	Hydrogen Bond Donor	Ethanol EE	Reference
**Alcohol-based systems**					
*Hibiscus sabdariffa* L.	MAE	5-*O*-Caffeoylshikimic acid	Maltose; Ethylene Glycol; 1,2-Propanediol;	1.24; 1.49; 1.54;	Alañón et al. [71]
		Chlorogenic acid	Maltose; 1,2-Propanediol; Ethylene Glycol;	0.76; 0.96; 0.98;	
		Chlorogenic acid quinone	Maltose; 1,2-Propanediol; Ethylene Glycol;	0.93; 1.06; 1.17;	
		Coumaroylquinic acid	Maltose; 1,2-Propanediol; Ethylene Glycol;	0.74; 0.95; 0.96;	
		Cryptochlorogenic acid	Maltose; 1,2-Propanediol; Ethylene Glycol;	0.78; 0.96; 1;	
*Rosmarinus officinalis* L.	UAE	Ferulic acid	Glycerol; 1,2-Propanediol; Glycerol; 1,4-Butanediol;	4.34; 5.45; 8.26; 9.99;	Barbieri et al. [56]
Wild Rice Powder	UAE	*p*-Hydroxybenzoic acid	Glycerol; 1,4-Butanediol;	2.47; 2.77;	Zeng et al. [61]
		Protocatechuic acid	1,4-Butanediol; Glycerol;	0.54; 0.59;	
*Rosmarinus officinalis L*.	UAE	Rosmarinic Acid	Glycerol; 1,2-Propanediol;	1.9; 2.7;	Barbieri et al. [56]
		Rutin	Glycerol	1.51	
Wild Rice Powder	UAE	Sinapic acid	Glycerol; 1,4-Butanediol;	0.89; 1.14;	Zeng et al. [61]
		Syringic acid	Glycerol; 1,4-Butanediol;	0.01; 2.44;	
*Cajanus cajan;* Orange Peel; Spent Coffee*; Rosmarinus officinalis* L.; Spent Coffee Grounds;	MAE; H/S; UAE;	Total Phenolics	Maltose; Sorbitol ^c^; Ethylene Glycol ^c^; Glycerol ^b,c^; Sorbitol ^c^; Ethylene Glycol ^c^; Ethylene Glycol ^b,c^; 1,4-Butanediol ^b,c^; Glycerol ^b,c^; Ethylene Glycol ^b,c^; Glycerol ^b,c^; Glycerol ^b,c^; Ethylene Glycol ^b,c^; 1,4-Butanediol ^b^; 1,4-Butanediol ^b^; 1,2-Propanediol ^b,c^; Glycerol ^b,c^; 1,3-Butanediol ^b^; 1,4-Butanediol ^b,c^; 1,6-Hexanediol ^b,c^; 1,2-Butanediol ^b^; 1,3-Butanediolb; 1,4-Butanediolb; 1,2-Butanediolb; 1,3-Propanediolb; 1,2-Propanediolb; 1,3-Propanediolb; 1,2-Propanediolb; 1,2-Propanediolb; 1,3-Propanediolb; 1,3-Butanediolb; 1,2-Butanediolb;	0.5; 0.54; 0.56; 0.59; 0.59; 0.72; 0.8; 0.81; 0.9; 0.98; 1.03; 1.11; 1.12; 1.14; 1.16; 1.22; 1.25; 1.35; 1.54; 1.64; 1.68; 1.81; 2.02; 2.44; 2.83; 3.8; 4.45; 4.48; 5.14; 6.25; 6.54; 7.47;	Wei et al. [72]; Ozturk et al. [70]; Yoo et al. [14]; Barbieri et al. [56]; Krisanti et al. [73];
Wild Rice Powder	UAE	Vanilin	1,4-Butanediol; Glycerol;	0.98; 1.17;	Zeng et al. [61]
		Vanillic acid	Glycerol; 1,4-Butanediol;	1.15; 1.56;	
*Hibiscus sabdariffa* L.	MAE	Neochlorogenic acid	Maltose; 1,2-Propanediol; Ethylene Glycol;	0.74; 0.93; 1;	Alañón et al. [71]
**Amide-based systems**					
*Hibiscus sabdariffa* L.	MAE	5-*O*-Caffeoylshikimic acid	Urea	1.62	Alañón et al. [71]
		Chlorogenic acid	Urea	0.94	
		Chlorogenic acid quinone	Urea	0.97	
		Coumaroylquinic acid	Urea	0.94	
		Cryptochlorogenic acid	Urea	1.03	
Wild Rice Powder	UAE	ferulic acid	Urea	7.00	Zeng et al. [61]
		*p*-Hydroxybenzoic acid	Urea	2.70	
		Protocatechuic acid	Urea	2.16	
		Sinapic acid	Urea	0.76	
		Syringic acid	Urea	2.66	
*Hibiscus sabdariffa* L.	MAE	Neochlorogenic acid	Urea	1.03	Alañón et al. [71]
Spent Coffee	UAE	Total Phenolics	Urea; Acetamide;	0.64; 1.16;	Yoo et al. [14]
Wild Rice Powder	UAE	Vanilin	Urea	1.63	Zeng et al. [61]
		Vanillic acid	Urea	1.17	
**Organic-acid-based systems**					
*Hibiscus sabdariffa* L.	*MAE*	5-*O*-Caffeoylshikimic acid	Oxalic Acid; Lactic Acid;	1.27; 1.82;	Alañón et al. [71]
		Chlorogenic acid	Lactic Acid; Oxalic Acid;	0.94; 0.94;	
		Chlorogenic acid quinone	Oxalic Acid; Lactic Acid;	0.92; 1.04;	
		Coumaroylquinic acid	Lactic Acid; Oxalic Acid;	0.99; 1.03;	
		Cryptochlorogenic acid	Lactic Acid; Oxalic Acid;	1.15; 1.18;	
		Neochlorogenic acid	Lactic Acid; Oxalic Acid;	0.94; 1.13;	
*Aegle marmelos*	UAE	Ascorbic Acid	Oxalic Acid ^b^; Oxalic Acid ^b^; Oxalic Acid ^b^;	0.64; 1.93; 2.17;	Saha et al. [65]
*Juglans regia* L.	H/S	Chlorogenic Acid	5-Phenylvaleric Acid; 3-Phenylpropionic Acid; 4-Phenylbutyric Acid; Citric Acid; Glutaric Acid; Acetic Acid; Phenylacetic Acid; Malic Acid; 3-Phenylpropionic Acid; Valeric Acid; Propanedioic Acid; Lactic Acid; Glycolic Acid; Butyric Acid; Propionic Acid;	0.43; 0.51; 0.78; 0.9; 0.9; 1; 1; 1.02; 1.1; 1.1; 1.14; 1.16; 1.27; 1.49; 1.59;	Vieira et al. [57]
Wild Rice Powder; *Rosmarinus officinalis L*.;	UAE	Ferulic acid	Lactic Acid; Oxalic Acid; Lactic Acid; Malic Acid;	5.06; 5.42; 5.96; 6.71;	Zeng et al. [61]; Barbieri et al. [56];
*Aegle marmelos*	UAE	Gallic Acid	Oxalic Acid ^b^; Oxalic Acid ^b^; Oxalic Acid ^b^;	0.73; 1.56; 1.65;	Saha et al. [65]
		*p*-Coumaric acid	Oxalic Acid ^b^; Oxalic Acid ^b^; Oxalic Acid ^b^;	0.66; 2.07; 2.14;	
Wild Rice Powder	UAE	*p*-Hydroxybenzoic acid	Lactic Acid; Malic Acid;	2.21; 2.79;	Zeng et al. [61]
*Aegle marmelos*; Wild Rice Powder;	UAE	Protocatechuic acid	Oxalic Acid ^b^; Malic Acid; Lactic Acid; Oxalic Acid ^b^; Oxalic Acid ^b^;	1.2; 1.56; 1.74; 2.03; 2.28;	Saha et al. [65]; Zeng et al. [61];
*Rosmarinus officinalis* L.	UAE	Rosmarinic Acid	Oxalic Acid; Lactic Acid;	1.9; 2.52;	Barbieri et al. [56]
		Rutin	Oxalic Acid; Lactic Acid;	2.02; 3.25;	
Wild Rice Powder	UAE	Sinapic acid	Lactic Acid; Malic Acid;	0.75; 0.88;	Saha et al. [65]
		Syringic acid	Lactic Acid; Malic Acid;	0.85; 2.6;	
*Cajanus cajan*	MAE	Total Phenolics	Lactic Acid ^b^; Malic Acid; Oxalic Acid; Citric Acid ^b^; Lactic Acid ^b^; Lactic Acid ^b^; Lactic Acid ^b^; Propanedioic Acid; Citric Acid ^b^; Lactic Acid ^b^; Lactic Acid ^b^; Lactic Acid ^b^; Levulinic Acid ^b^; Levulinic Acid ^b^; Lactic Acid ^b^; Levulinic Acid ^b^; Lactic Acid ^b^; Lactic Acid ^b^;	0.49; 0.63; 0.92; 0.99; 1.03; 1.18; 1.21; 1.25; 1.26; 1.27; 1.47; 1.59; 2.47; 2.8; 3.38; 3.6; 3.93; 5.69;	Wei et al. [74]
Wild Rice Powder	UAE	Vanilin	Lactic Acid	1.36	Zeng et al. [61]
**Sugar-based systems**					
*Hibiscus sabdariffa* L.	*MAE*	5-*O*-Caffeoylshikimic acid	Fructose; Glucose;	1.36; 1.38;	Alañón et al. [63]
		Chlorogenic acid	Glucose; Fructose;	0.84; 0.86;	
		Chlorogenic acid quinone	Fructose; Glucose;	0.97; 1.06;	
		Coumaroylquinic acid	Glucose; Fructose;	0.83; 0.85;	
		Cryptochlorogenic acid	Fructose; Glucose;	0.87; 0.87;	
*Rosmarinus officinalis L*.	UAE	Ferulic acid	Glucose; Fructose;	0.61; 9.38;	Barbieri et al. [56]
Wild Rice Powder	UAE	*p*-Hydroxybenzoic acid	Glucose; Fructose;	2.02; 2.3;	Zeng et al. [61]
		Protocatechuic acid	Fructose; Glucose;	0.83; 1.33;	
		Sinapic acid	Glucose; Fructose;	0.46; 1.05;	
		Syringic acid	Fructose; Glucose;	1.43; 1.61;	
Spent Coffee	UAE	Total Phenolics	Glucose ^b^; Sucrose ^b^; Sucrose ^b^; Glucose ^b^; Xylose; Fructose;	0.35; 0.51; 0.71; 0.88; 1.08; 2.08;	Yoo et al. [14]
Wild Rice Powder	UAE	Vanilin	Fructose; Glucose;	0.86; 1.1;	Zeng et al. [61]
		Vanillic acid	Glucose; Fructose;	1.57; 1.75;	

^b^ represents a different HBD/HBA ratio; ^c^ represents different natural matrices.

**Table 6:** Correlation between the natural matrix, extraction method, and DES based on ammonium salts (choline chloride and betaine) and the effect of different HBDs on the extraction efficiency of different phenolic acids compared with ethanol extractions.

Natural Matrix	Extraction Method	Extracted Compound	Hydrogen Bond Donor	Methanol EE	Reference
**Alcohol-based systems**					
*Artemisia argyi*	UAE	3,4-Di-*O*-Cafeoylquinic Acid	Glycerol; Ethylene Glycol;	0.66; 0.77;	Duan et al. [75]
		3,5-Di-*O*-Cafeoylquinic Acid	Glycerol; Ethylene Glycol;	0.65; 0.96;	
		3-Caffeoylquinic Acid	Glycerol; Ethylene Glycol;	0.70; 0.86;	
		4,5-Di-*O*-Cafeoylquinic Acid	Glycerol; Ethylene Glycol;	0.64; 0.84;	
*Camelia sinesis* Seed Oil	H/S	Benzoic Acid	Propilene Glycol; Ethylene Glycol; Glycerol; Xylitol;	0.63; 0.90; 1.07; 1.19;	Wang et al. [67]
		Caffeic Acid	Xylitol; Propilene Glycol; Ethylene Glycol; Glycerol;	1.32; 1.88; 1.97; 4.54;	
		Cinnamic Acid	Xylitol; Glycerol; Ethylene Glycol; Propilene Glycol;	0.67; 1.08; 1.29; 2.18;	
		Ferulic Acid	Glycerol; Ethylene Glycol; Propilene Glycol; Xylitol;	0.54; 0.59; 0.74; 2.65;	
		Gallic Acid	Propilene Glycol; Glycerol; Xylitol; Propanedioic Acid; Ethylene Glycol;	0.59; 0.96; 1.12; 0.75; 1.19;	
		*p*-Coumaric acid	Xylitol; Ethylene Glycol; Propilene Glycol; Glycerol;	0.48; 0.81; 0.92; 1.26;	
		Phthalic Acid	Glycerol; Propilene Glycol; Ethylene Glycol; Xylitol;	1.25; 1.51; 1.92; 2.07;	
		*p*-Hydrobenzoic Acid	Xylitol; Propilene Glycol; Ethylene Glycol; Glycerol;	0.31; 0.72; 0.74; 0.97;	
		*p*-Hydroxyphenylacetic Acid	Propilene Glycol; Glycerol; Ethylene Glycol; Xylitol;	1.38; 1.69; 3.24; 4.36;	
		Protocatechuic Acid	Propilene Glycol; Xylitol; Ethylene Glycol; Glycerol;	0.19; 0.32; 0.7; 1.6;	
Prunella vulgaris	H/S	Rosmairic Acid	2,3-Butanediol ^b^; 1,4-Butanediol ^b^; 1,4-Butanediol ^b^; 1,3-Butanediol ^b^; 1,3-Butanediol ^b^; 1,4-Butanediol ^b^; 1,3-Butanediol ^b^; 2,3-Butanediol ^b^; 2,3-Butanediol ^b^; 2,3-Butanediol ^b^; 1,3-Butanediol ^b^; 1,4-Butanediol ^b^; 1,4-Butanediol ^b^; 1,2-Propylene glycol ^b^; 2,3-Butanediol ^b^; 1,3-Butanediol ^b^; Glycerol ^b^; Glycerol ^b^; Glycerol ^b^; 1,2-Propylene glycol ^b^; Glycerol ^b^; 1,2-Propylene glycol ^b^; Glycerol ^b^; 1,2-Propylene glycol ^b^; Ethylene Glycol ^b^; 1,2-Propylene glycol ^b^; Ethylene Glycol ^b^; Ethylene Glycol ^b^; Ethylene Glycol ^b^; Ethylene Glycol ^b^;	0.36; 0.43; 0.43; 0.45; 0.45; 0.46; 0.49; 0.49; 0.52; 0.53; 0.55; 0.55; 0.58; 0.61; 0.62; 0.63; 0.63; 0.63; 0.68; 0.68; 0.69; 0.7; 0.74; 0.78; 0.79; 0.79; 0.81; 0.85; 0.87; 0.91;	Xia et al. [76]
		Salviaflaside	2,3-Butanediol ^b^; 1,3-Butanediol ^b^; 1,4-Butanediol ^b^; 1,3-Butanediol ^b^; 1,4-Butanediol ^b^; 1,3-Butanediol ^b^; 1,4-Butanediol ^b^; 1,3-Butanediol ^b^; 1,4-Butanediol ^b^; 2,3-Butanediol ^b^; 2,3-Butanediol ^b^; 2,3-Butanediol ^b^; 1,3-Butanediol ^b^; 1,2-Propylene glycol ^b^; 1,4-Butanediol ^b^; Glycerol ^b^; 2,3-Butanediol ^b^; Glycerol ^b^; Glycerol ^b^; Glycerol ^b^; 1,2-Propylene glycol ^b^; Glycerol ^b^; 1,2-Propylene glycol ^b^; 1,2-Propylene glycol ^b^; Ethylene Glycol ^b^; 1,2-Propylene glycol ^b^; Ethylene Glycol ^b^; Ethylene Glycol ^b^; Ethylene Glycol ^b^; Ethylene Glycol ^b^;	0.46; 0.51; 0.51; 0.54; 0.57; 0.6; 0.60; 0.68; 0.69; 0.69; 0.70; 0.70; 0.74; 0.76; 0.77; 0.78; 0.79; 0.8; 0.81; 0.82; 0.82; 0.83; 0.85; 0.86; 0.87; 0.87; 0.88; 0.90; 0.91; 0.98;	
*Camelia sinesis* Seed Oil	H/S	Sinapic Acid	Glycerol; Ethylene Glycol; Xylitol; Propilene Glycol;	0.56; 0.58; 0.77; 1.06;	Wang et al. [67]
Spent Coffee	UAE	Total Phenolics	Sorbitol; Glycerol; Ethylene Glycol; 1,4-Butanediol; 1,6-Hexanediol;	0.58; 1.1; 1.11; 1.53; 1.62;	Yoo et al. [14]
*Camelia sinesis* Seed Oil	H/S	Vanillic Acid	Glycerol; Xylitol; Ethylene Glycol; Propilene Glycol;	0.82; 1.32; 1.09; 1.07;	Wang et al. [67]
**Amide-based systems**					
*Artemisia argyi*	UAE	3,4-Di-*O*-Cafeoylquinic Acid	Urea	0.97	Duan et al. [75]
		3,5-Di-*O*-Cafeoylquinic Acid	Urea	0.84	
		3-Caffeoylquinic Acid	Urea	0.92	
		4,5-Di-*O*-Cafeoylquinic Acid	Urea	0.77	
Spent Coffee; *Aronia melanocarpa*	UAE	Total Phenolics	Urea ^b,c^; Urea ^b,c^; Urea ^b,c^; Acetamide;	0.63; 0.64; 0.74; 1.15;	Yoo et al. [14]; Razboršek et al. [77]
**Organic-acid-based systems**					
*Artemisia argyi*	UAE	3,4-Di-*O*-Cafeoylquinic Acid	Glutaric Acid; Malic Acid; Propanedioic Acid;	0.72; 0.89; 0.89;	Duan et al. [75]
		3,5-Di-*O*-Cafeoylquinic Acid	Glutaric Acid; Propanedioic Acid; Malic Acid;	0.73; 0.89; 0.94;	
		3-Caffeoylquinic Acid	Glutaric Acid; Propanedioic Acid; Malic Acid;	0.73; 0.89; 0.94;	
		4,5-Di-*O*-Cafeoylquinic Acid	Glutaric Acid; Propanedioic Acid; Malic Acid;	0.7; 0.86; 0.93;	
*Camelia sinesis* Seed Oil	H/S	Benzoic Acid	Propanedioic Acid	0.22	Wang et al. [67]
		Cinnamic Acid	Propanedioic Acid	0.7	
		Ferulic Acid	Propanedioic Acid	0.31	
		Gallic Acid	Propanedioic Acid	0.75	
		*p*-Coumaric acid	Propanedioic Acid	0.01	
		Phthalic Acid	Propanedioic Acid	0.4	
		*p*-Hydrobenzoic Acid	Propanedioic Acid	0.22	
		*p*-Hydroxyphenylacetic Acid	Propanedioic Acid	1.39	
		Protocatechuic Acid	Propanedioic Acid	0.07	
		Sinapic Acid	Propanedioic Acid	0.19	
*Aronia melanocarpa*	UAE	Total Phenolics	Lactic Acid; Lactic Acid; Lactic Acid; Lactic Acid; Propanedioic Acid; Citric Acid;	0.59; 0.63; 0.88; 1.02; 1.24; 1.25;	Razboršek et al. [77]
*Camelia sinesis* Seed Oil	H/S	Vanillic Acid	Propanedioic Acid	0.36	Wang et al. [67]
**Sugar-based systems**					
Spent Coffee; *Aronia melanocarpa*;	UAE	Total Phenolics	Glucose; Sucrose; Glucose; Glucose;	0.34; 0.51; 0.87; 0.96;	Yoo et al. [14]; Razboršek et al. [77];

^b^ represents a different HBD/HBA ratio; ^c^ represents different natural matrices.
